# Supplementary material for: β-cell-specific deletion of PFKFB3 restores cell fitness competition and physiological replication under diabetogenic stress
Source: Commun Biol. 2022 Mar 22;5:248. doi: 10.1038/s42003-022-03209-y (PMC8941137; doi:10.1038/s42003-022-03209-y)
Supplement: Supplementary file 2 — Supplementary Information [file 42003_2022_3209_MOESM2_ESM.pdf]

β-cell-specific deletion of *PFKFB3* restores cell fitness competition and physiological replication under diabetogenic stress

Jie Min[1,2], Feiyang Ma[3], Berfin Seyran[1], Matteo Pellegrini[3], Oppel Greeff[4], Salvador Moncada[5] and Slavica Tudzarova[1*]

Supplementary Information:
Supplementary Figures 1-17
Supplementary Tables 1-8
Supplementary Figure and Table Legends

Supplementary Figure 1

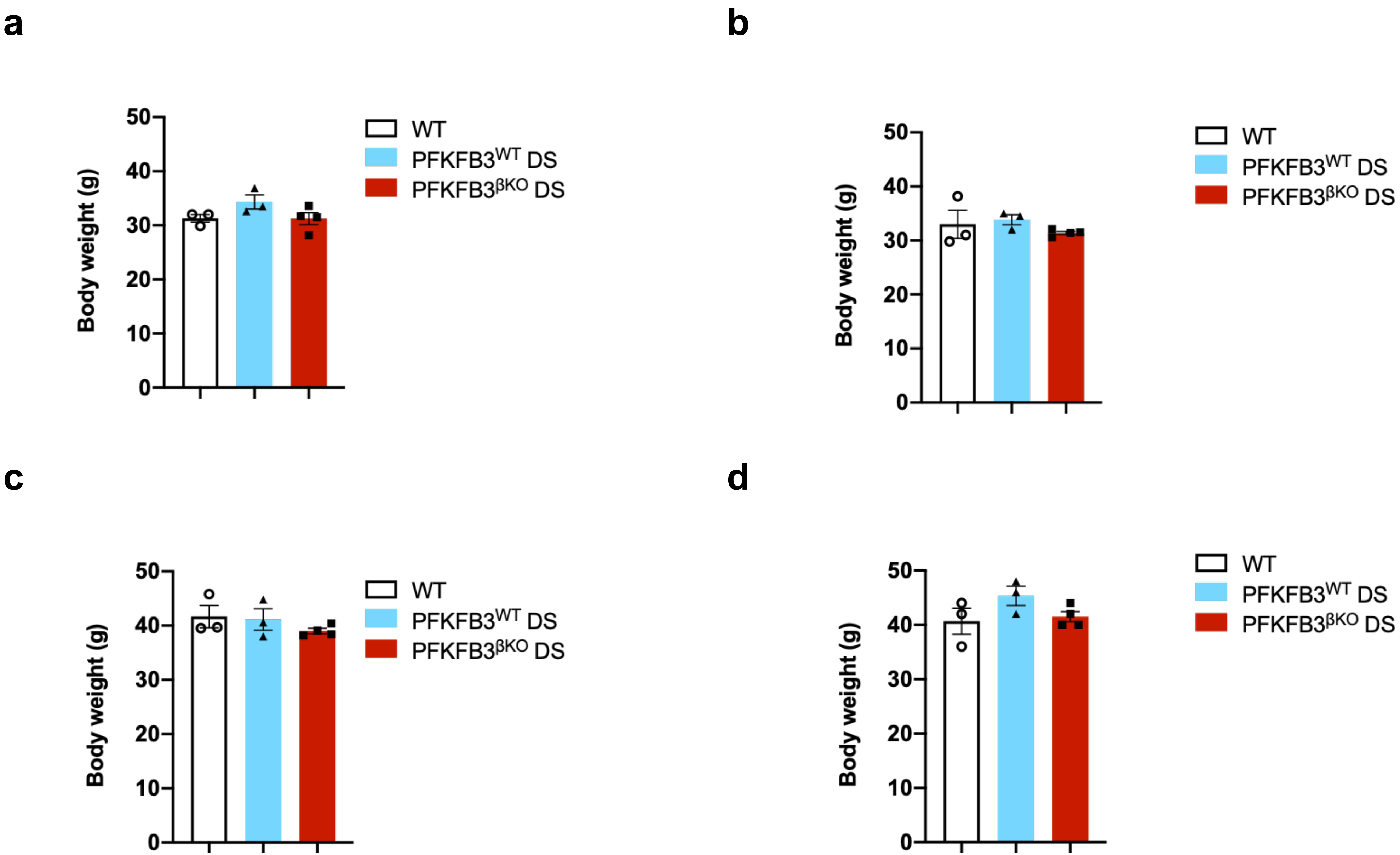

Supplementary Figure 2

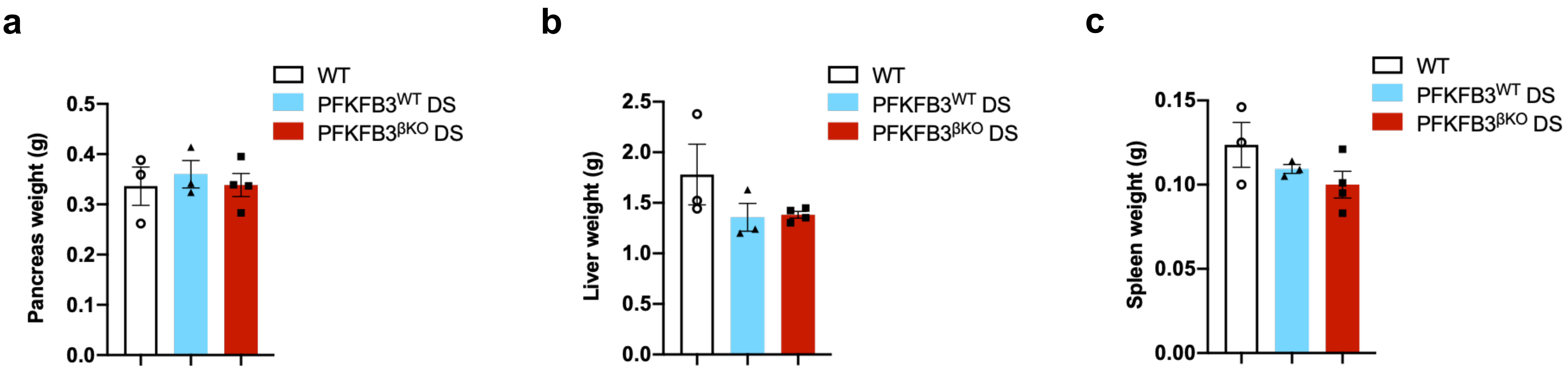

Supplementary Figure 3

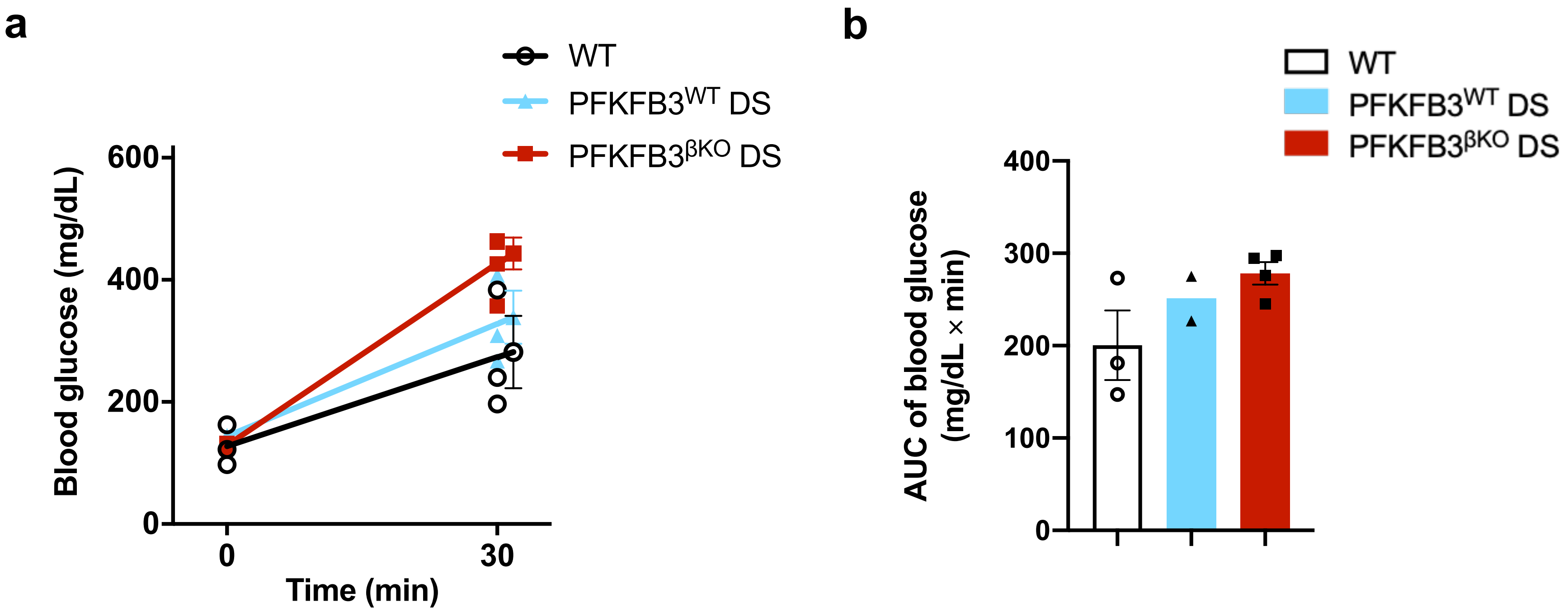

Supplementary Figure 4

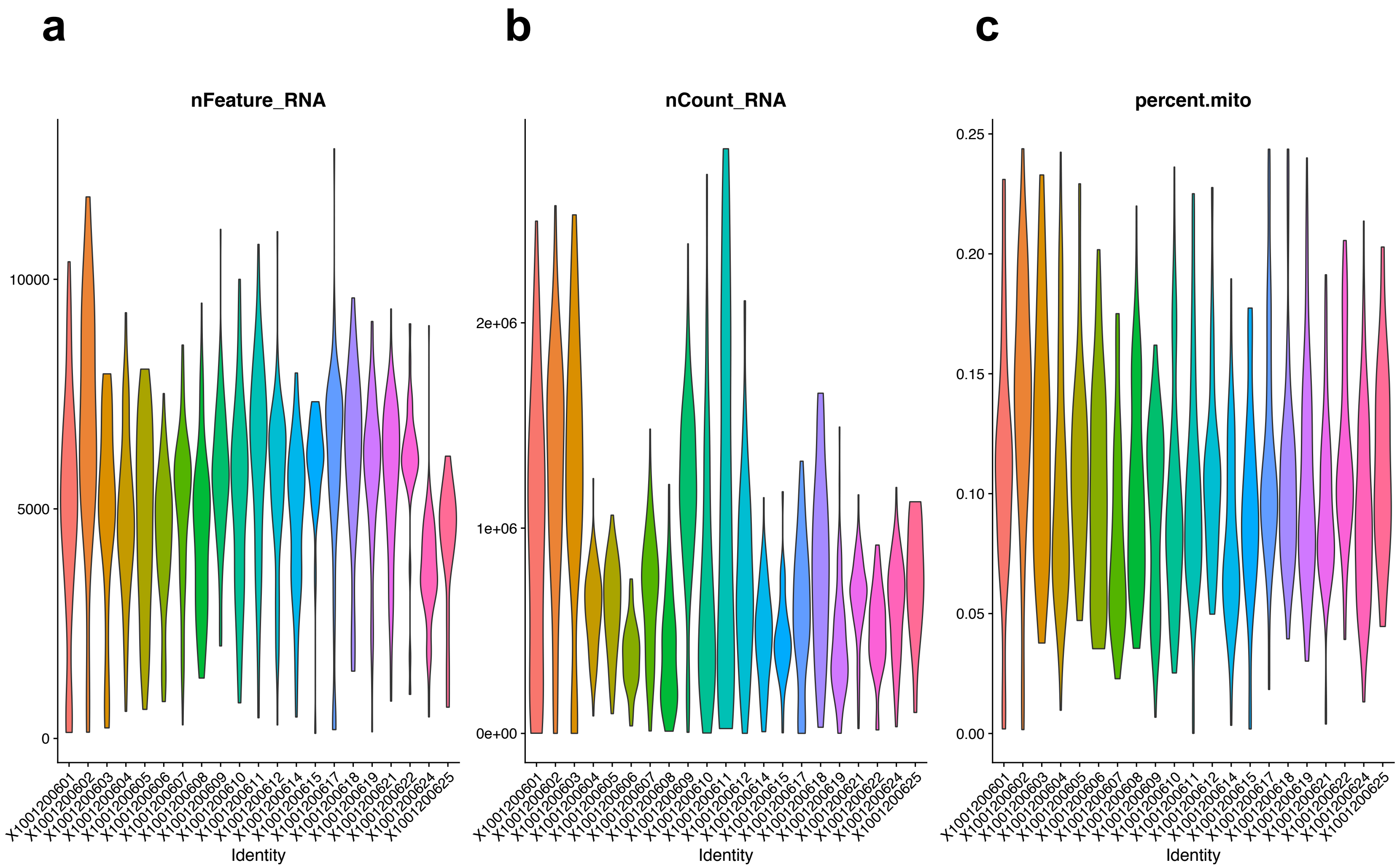

Supplementary Figure 5

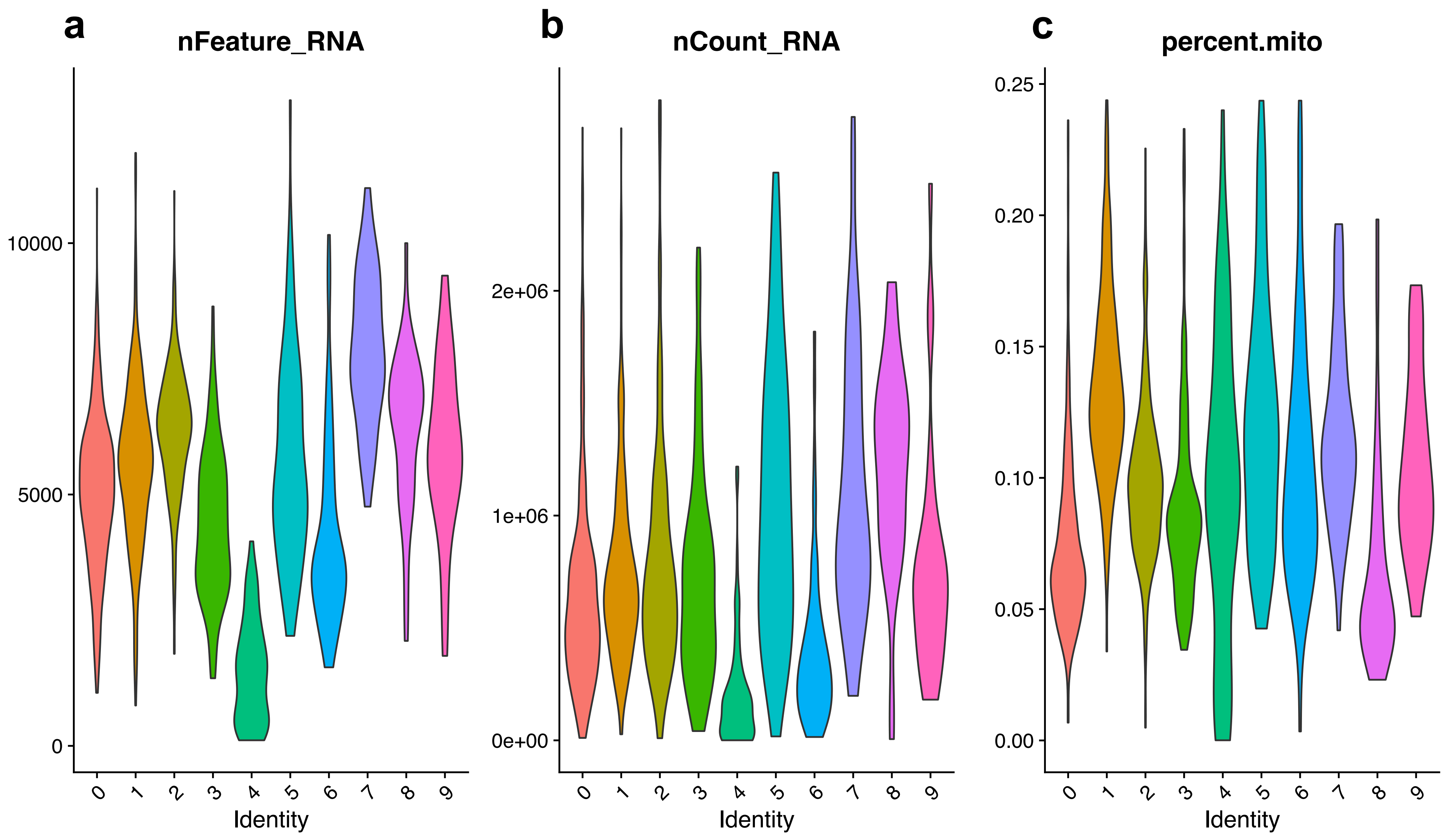

Supplementary Figure 6

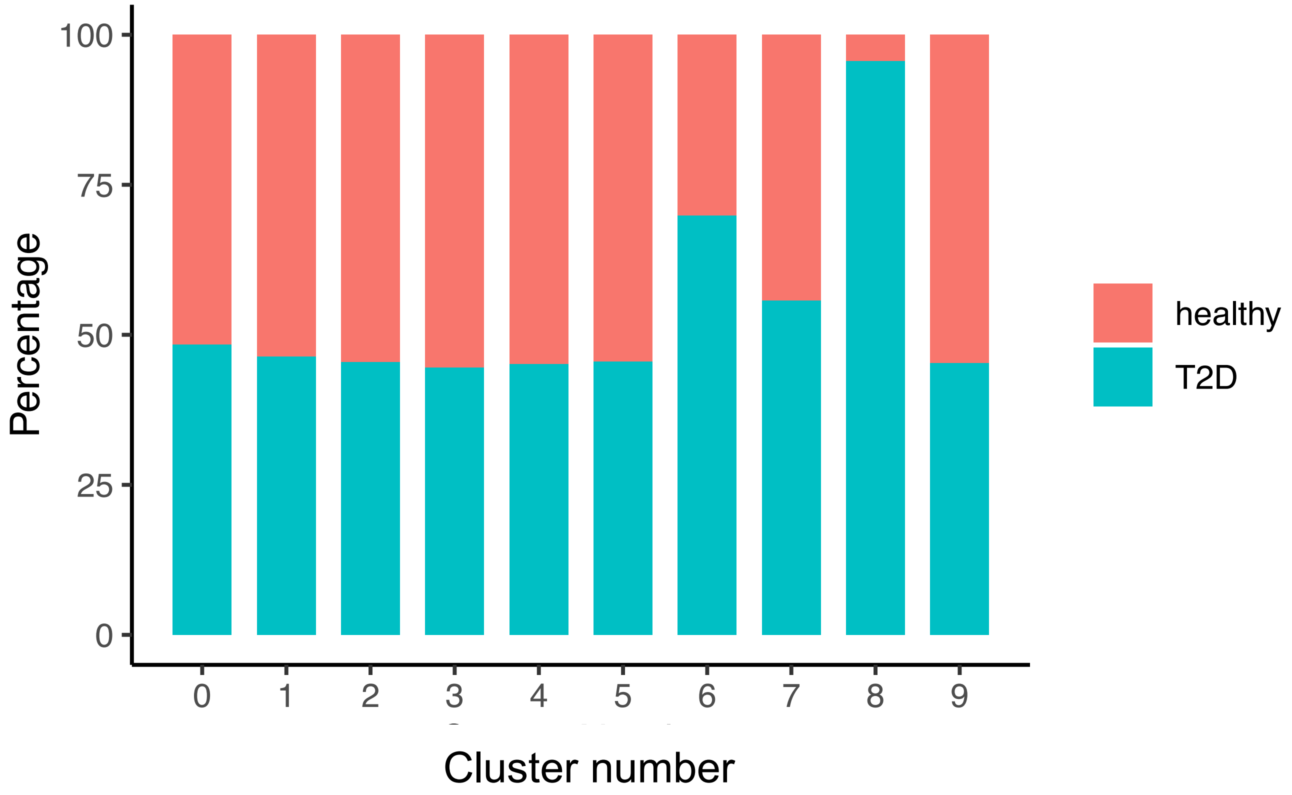

Supplementary Figure 7

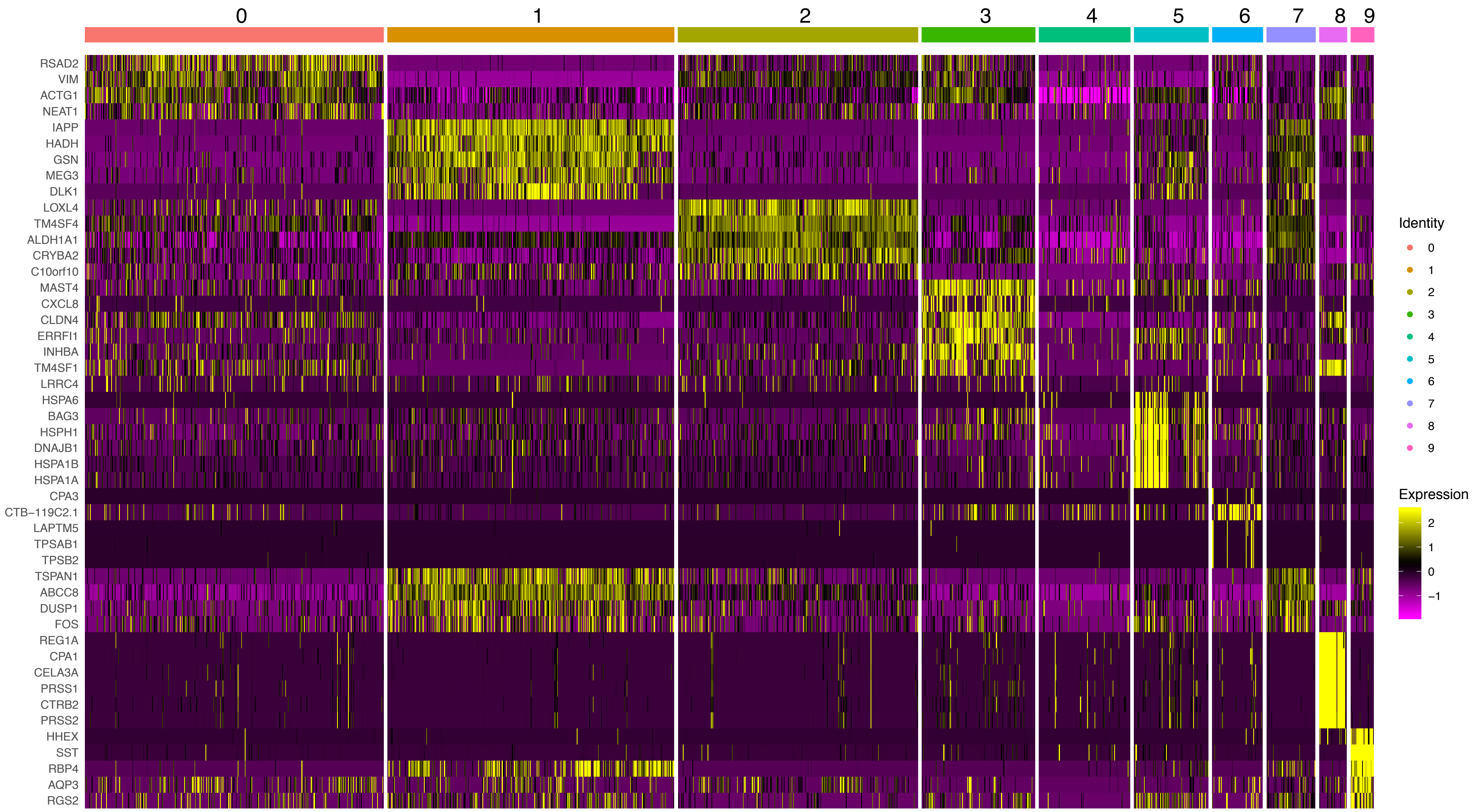

Supplementary Figure 8

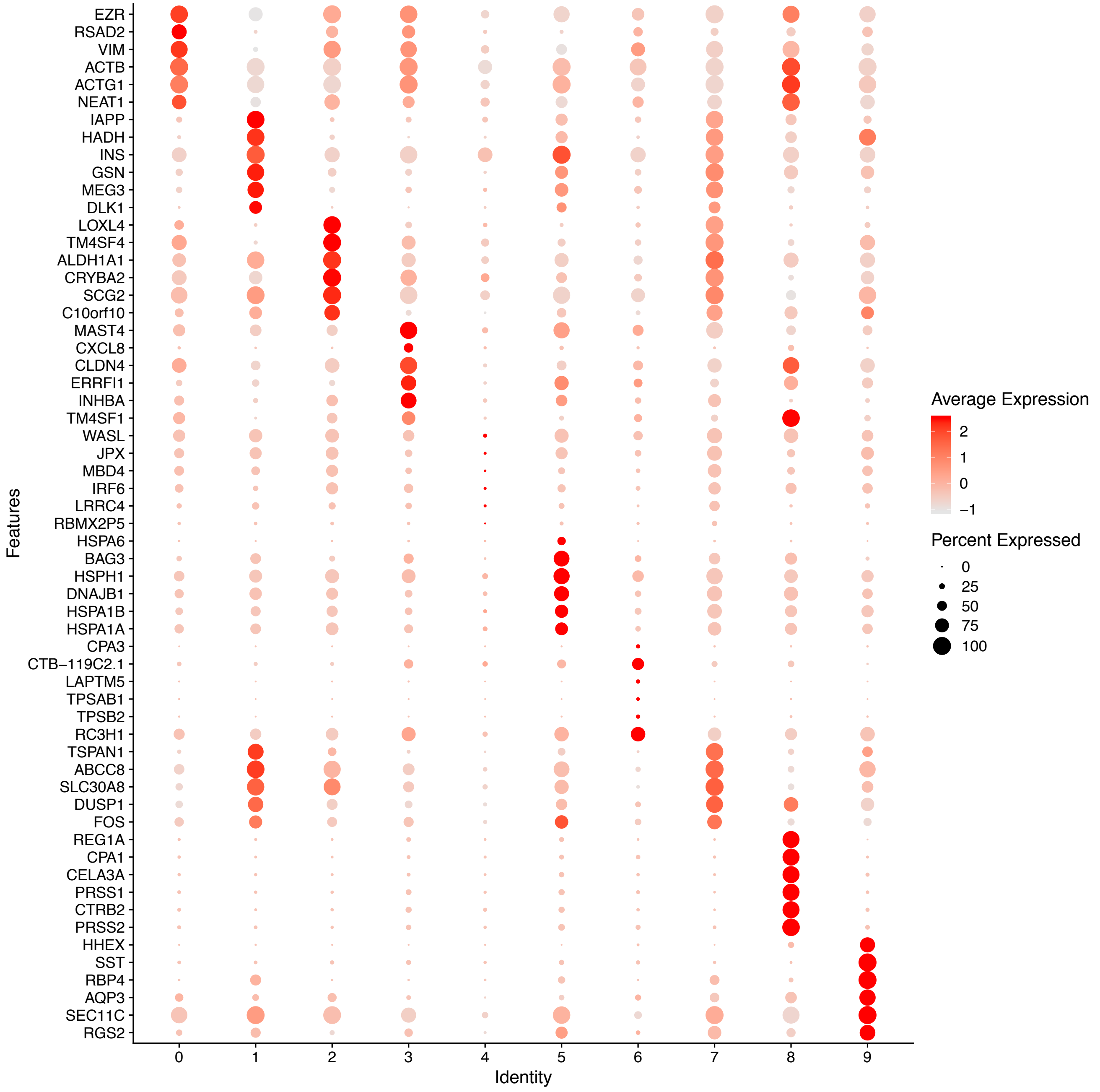

Supplementary Figure 9

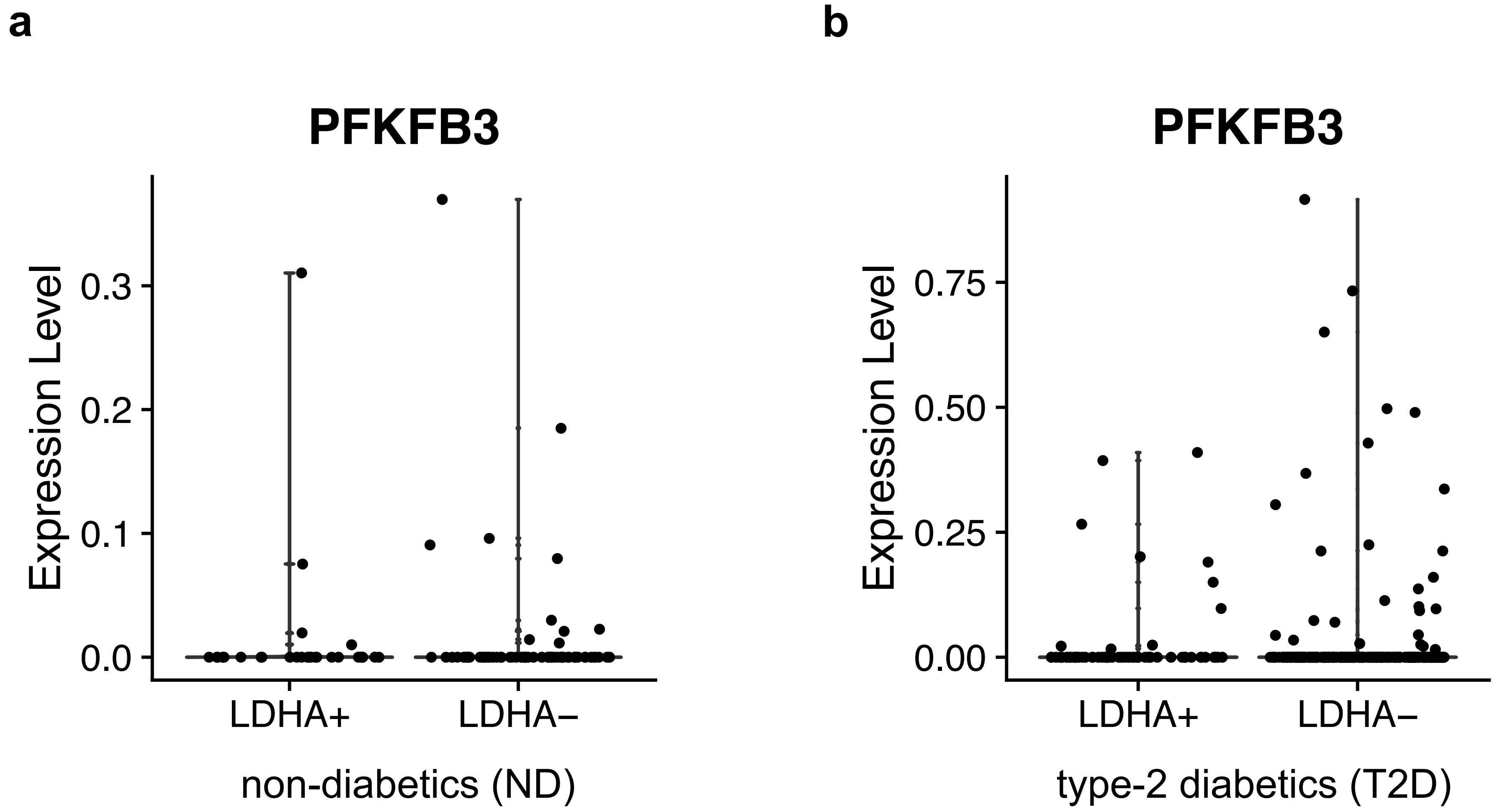

Supplementary Figure 10

a

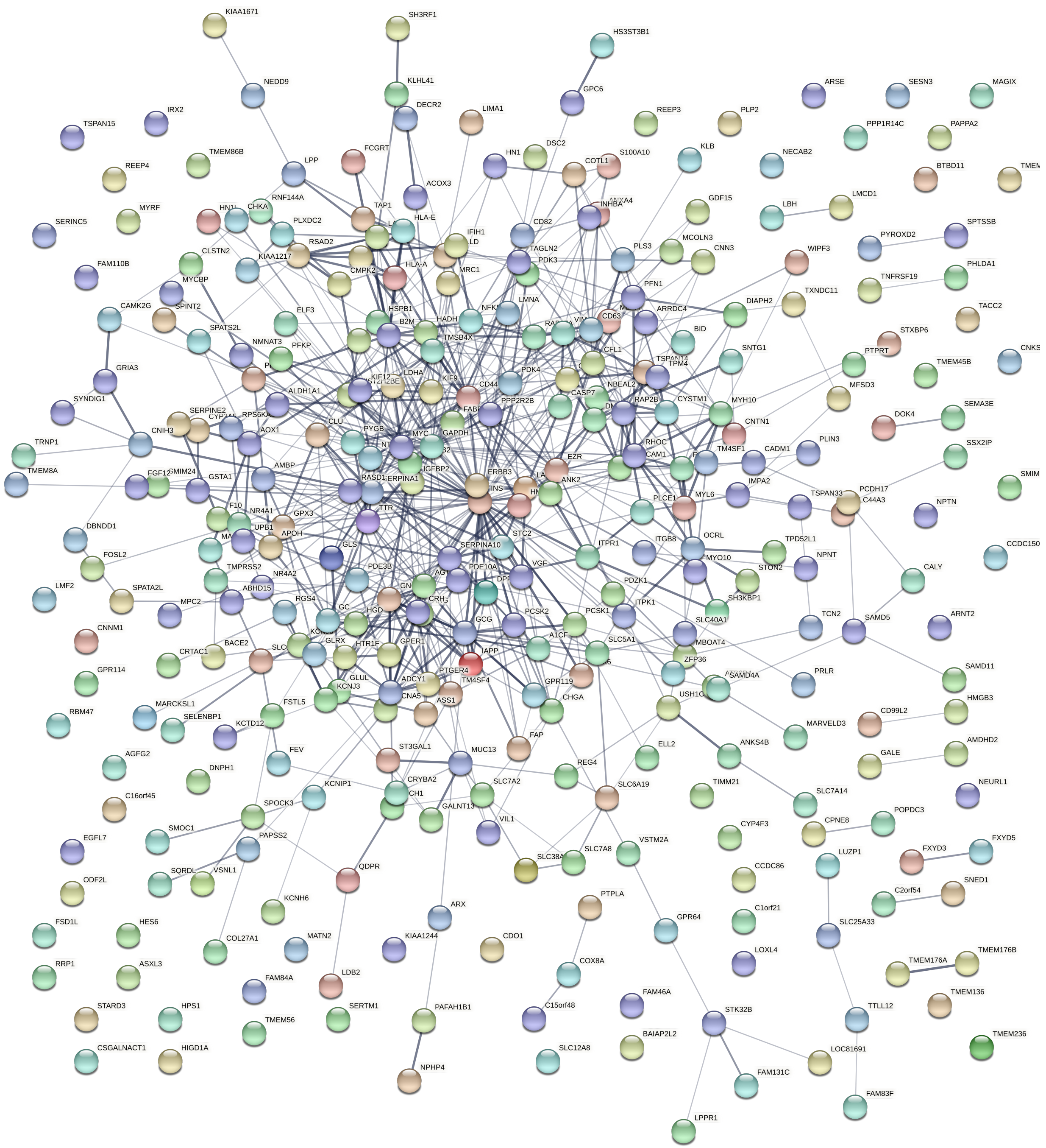

b

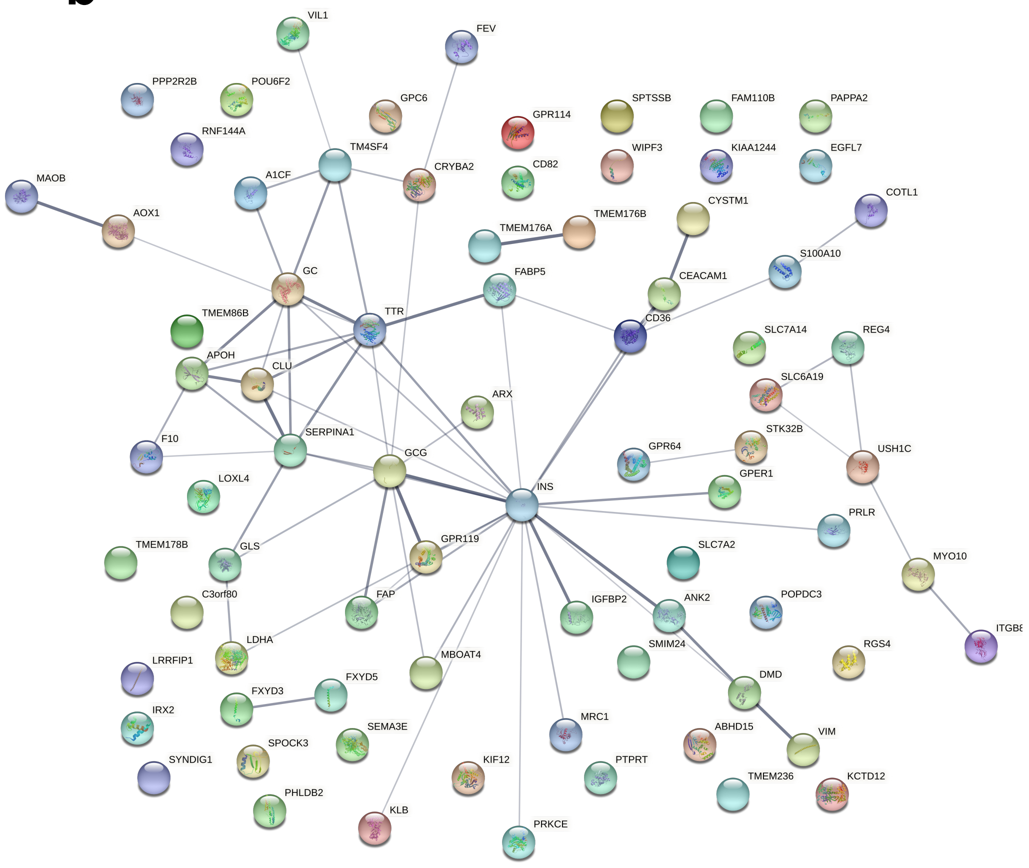

Supplementary Figure 11

a

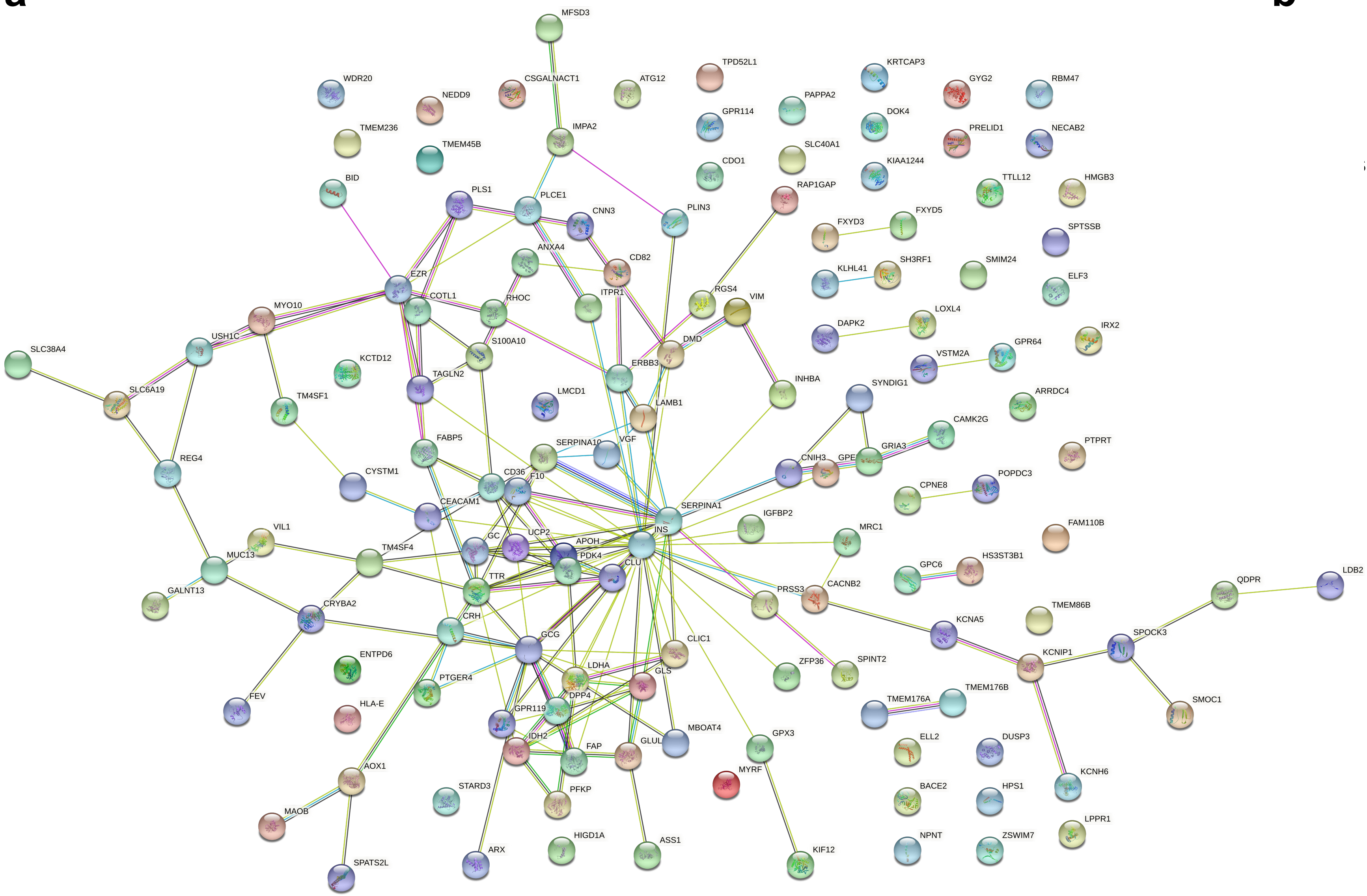

b

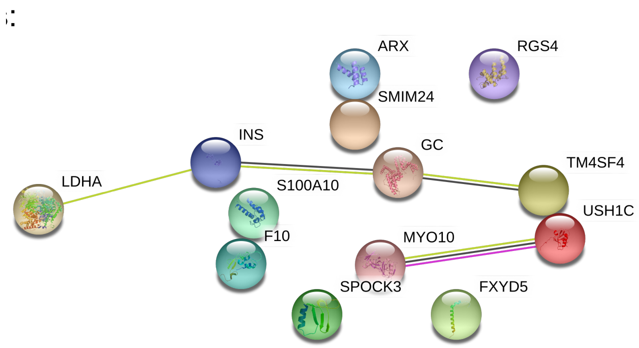

Supplementary Figure 12

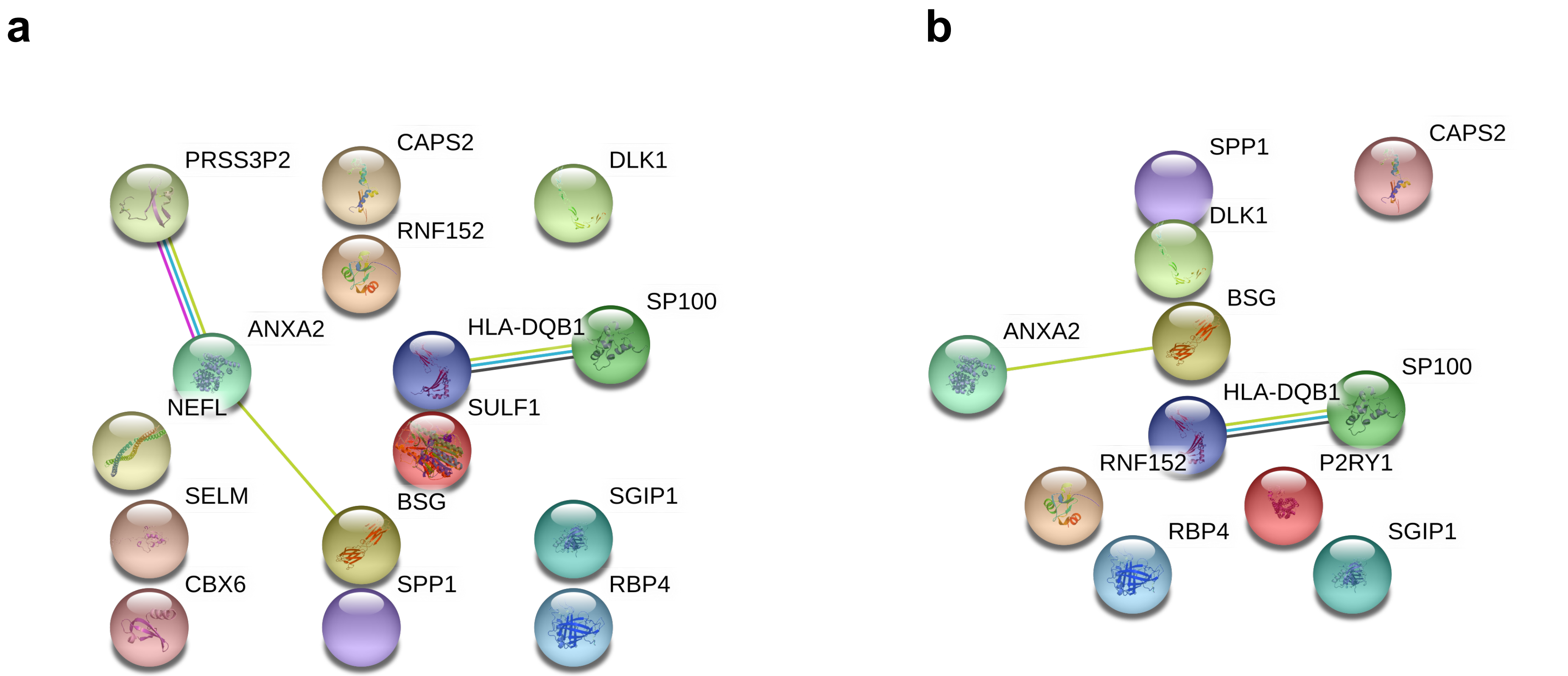

Supplementary Figure 13

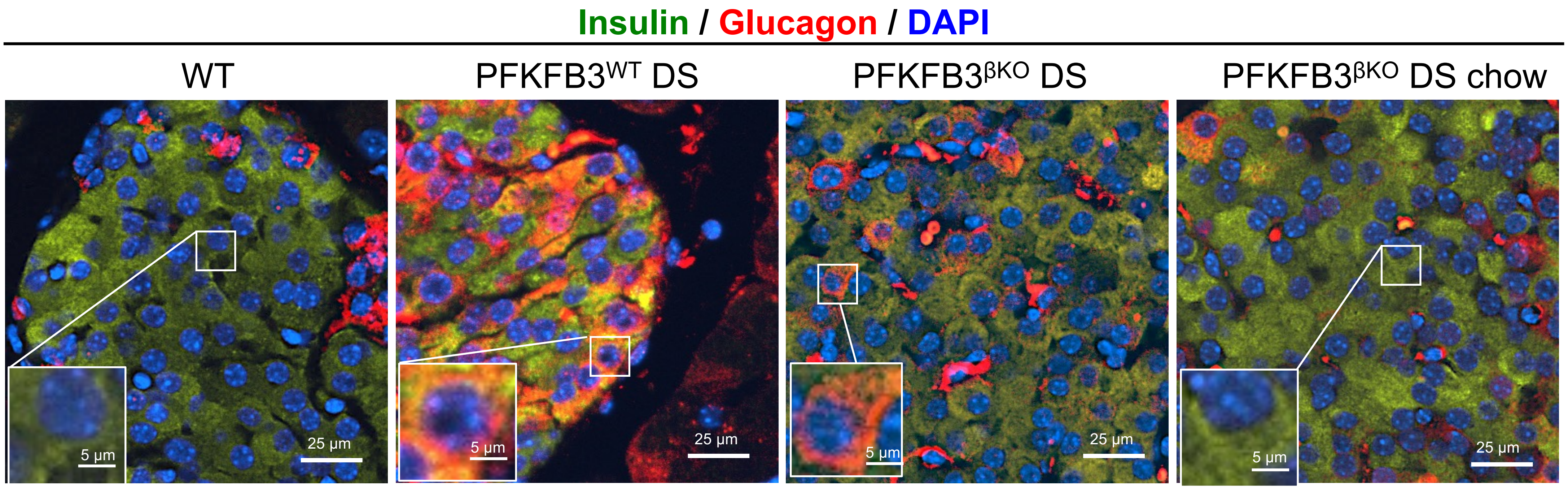

Supplementary Figure 14

a

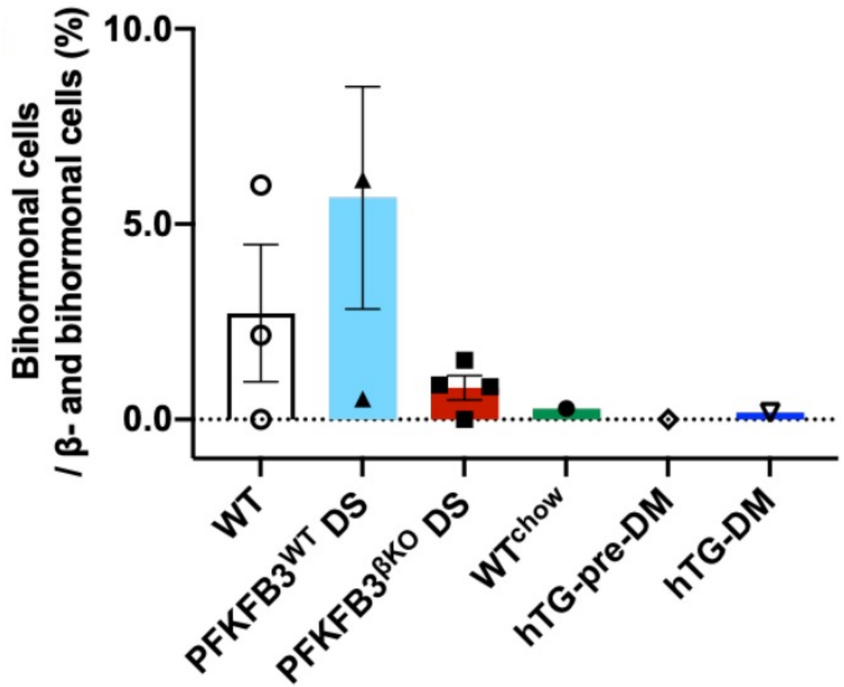

b

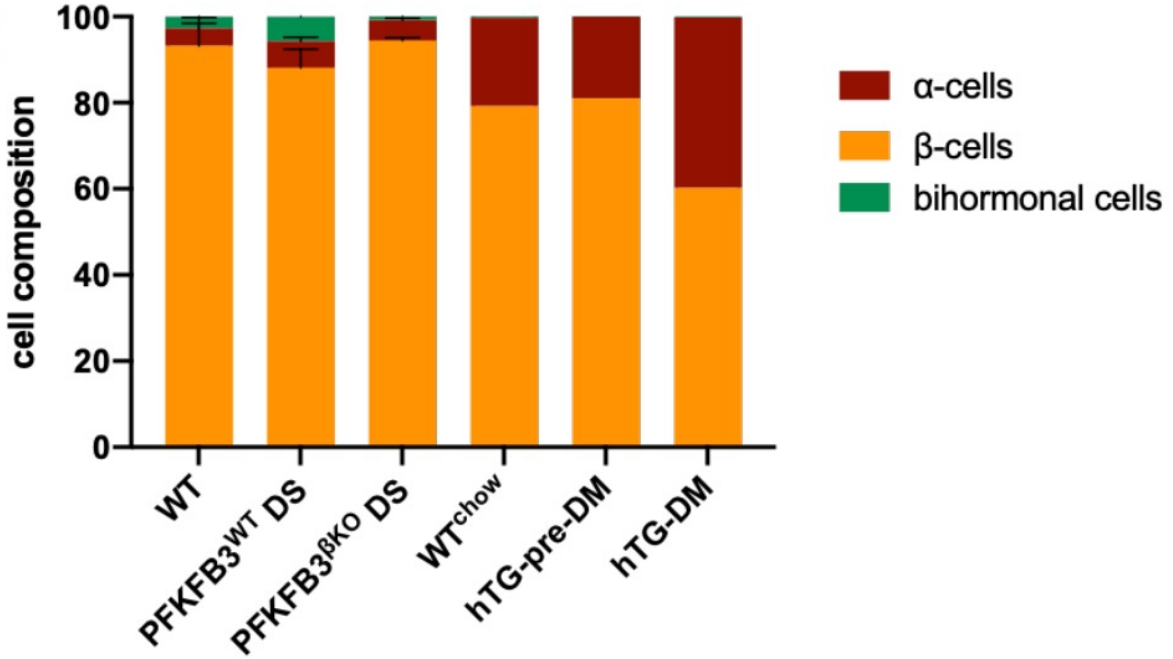

Supplementary Figure 15

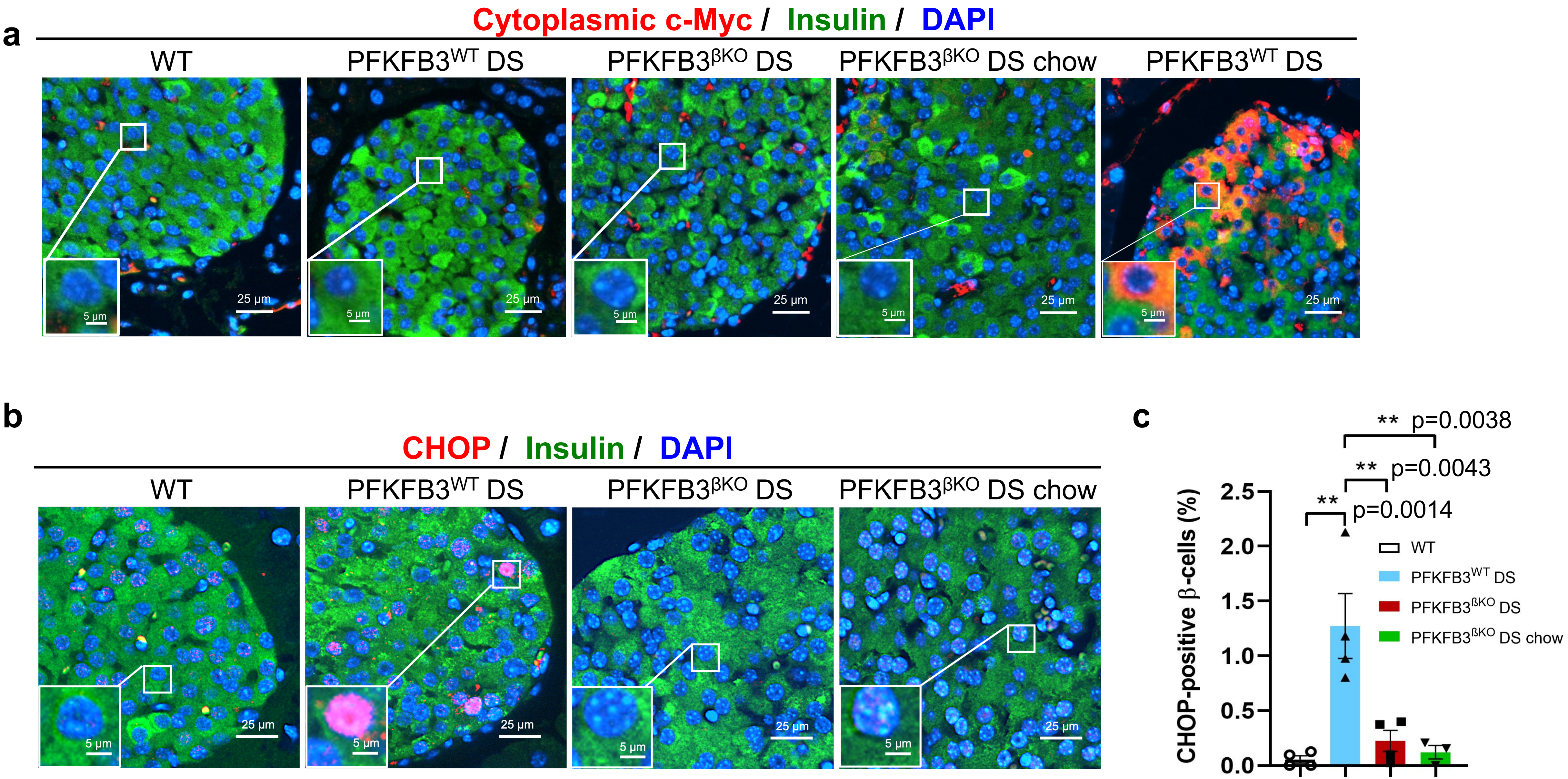

Supplementary Figure 16

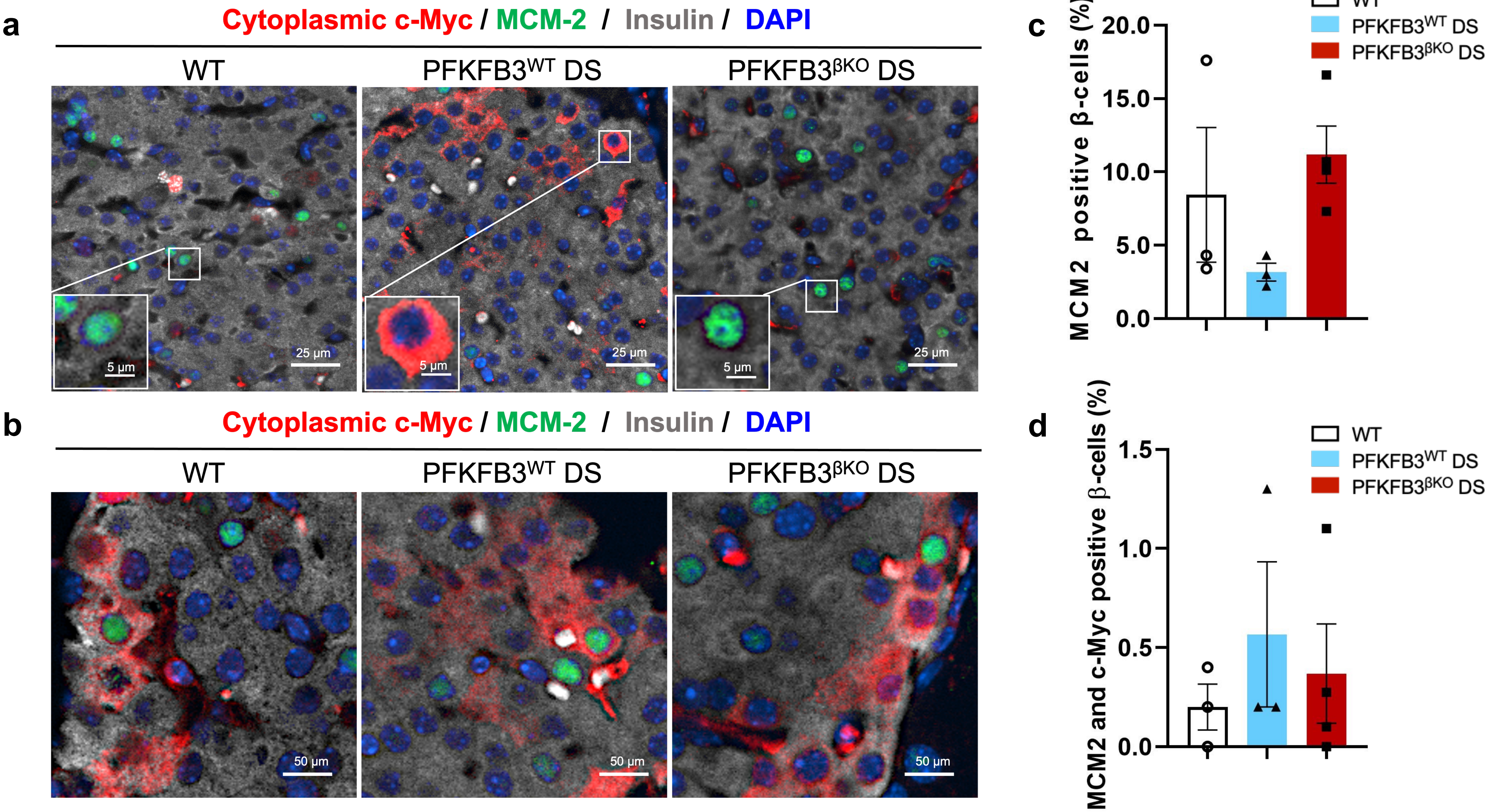

Supplementary Figure 17 a-p

a. Supporting data for Figure 1a

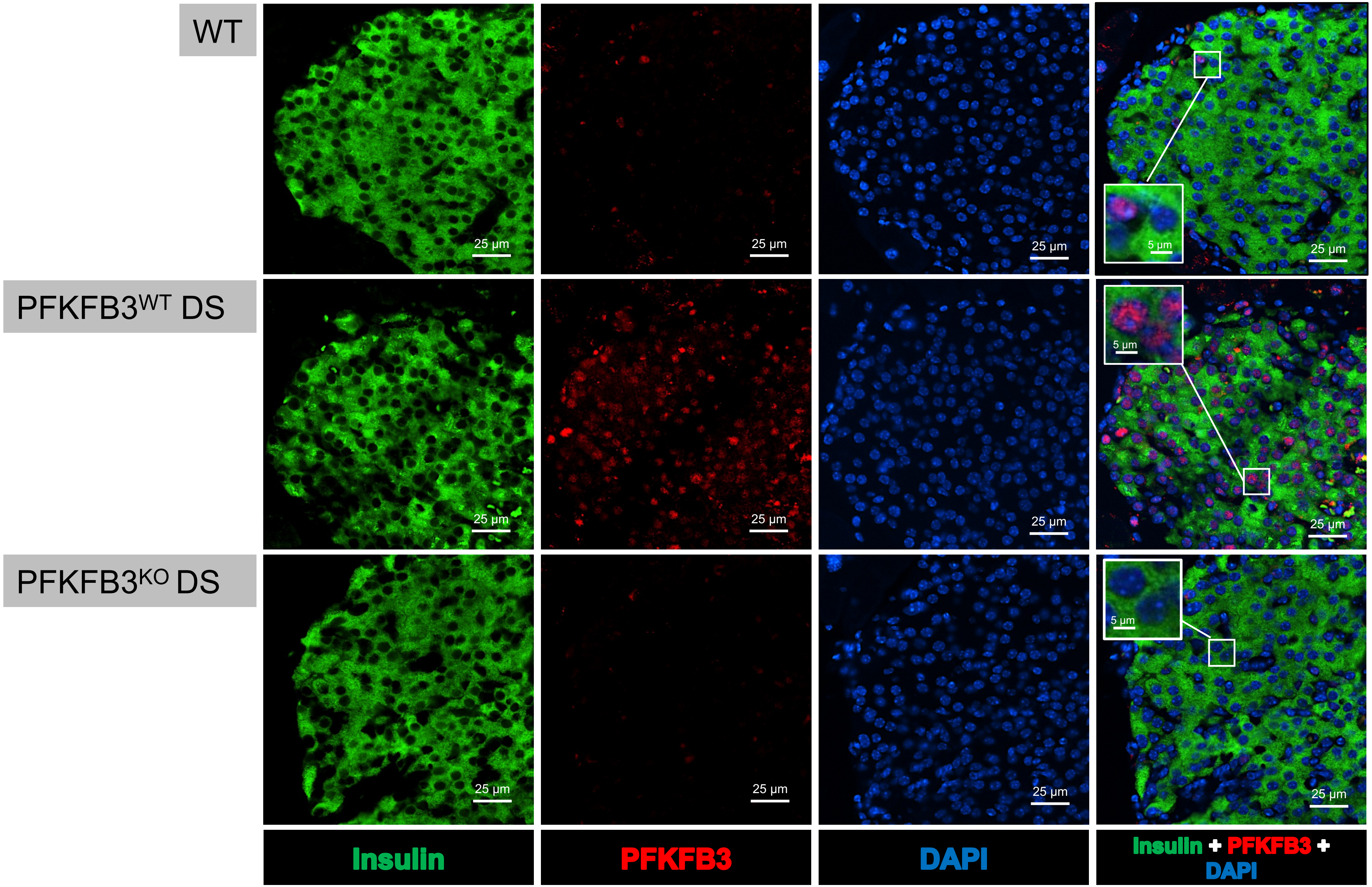

b. Supporting data for Figure 1a

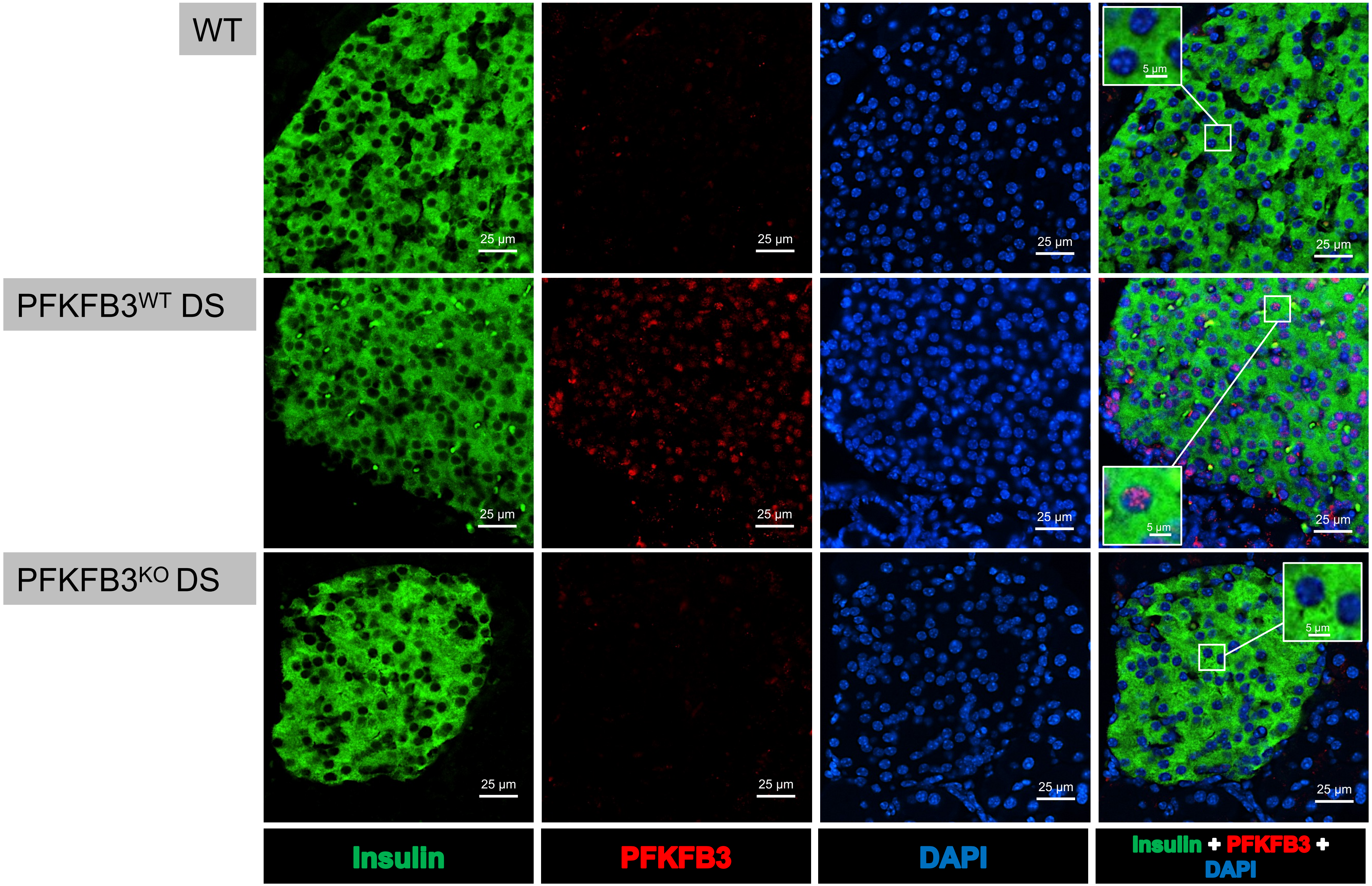

c. Supporting data for Figure 1c

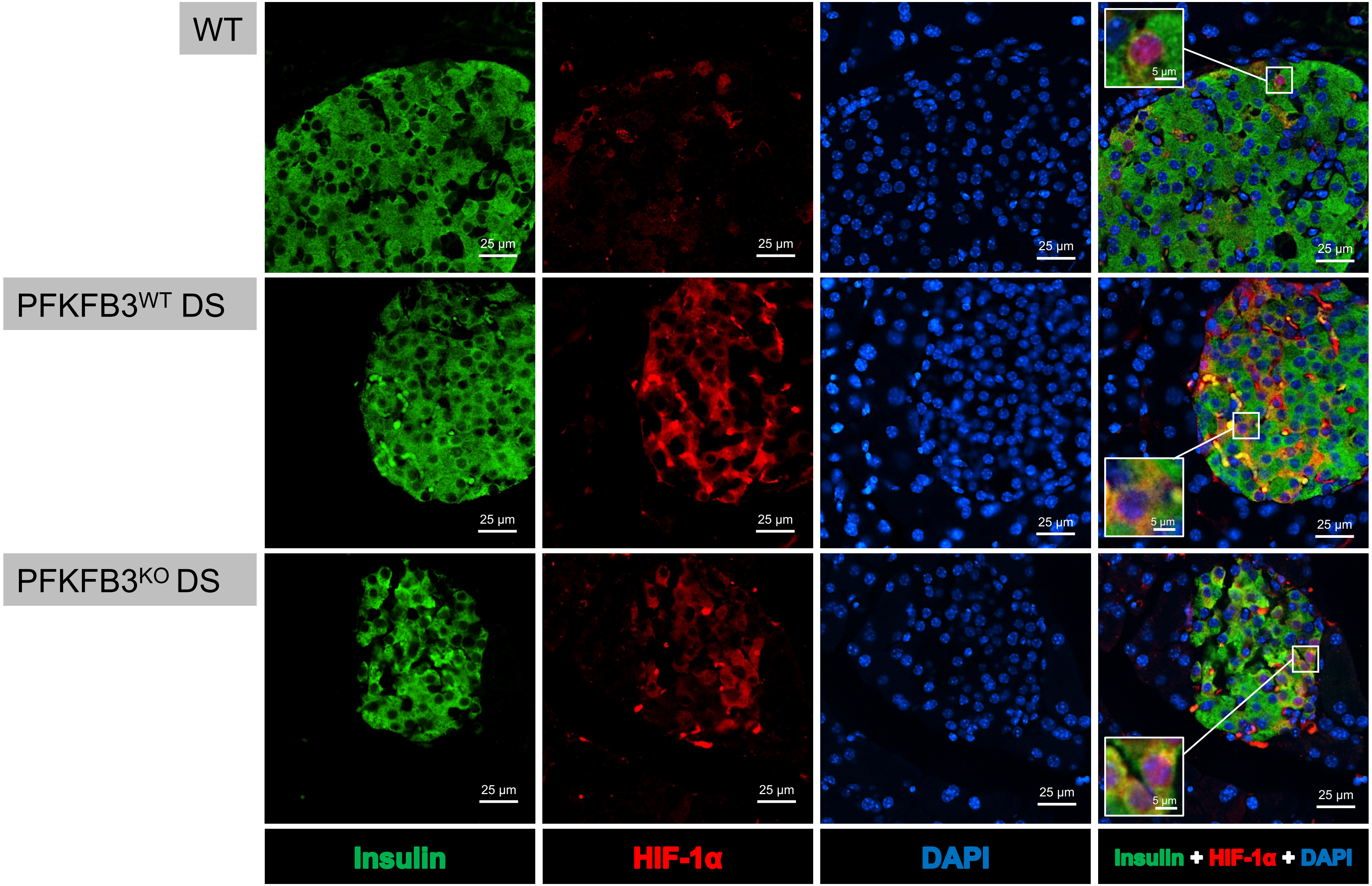

d. Supporting data for Figure 1c

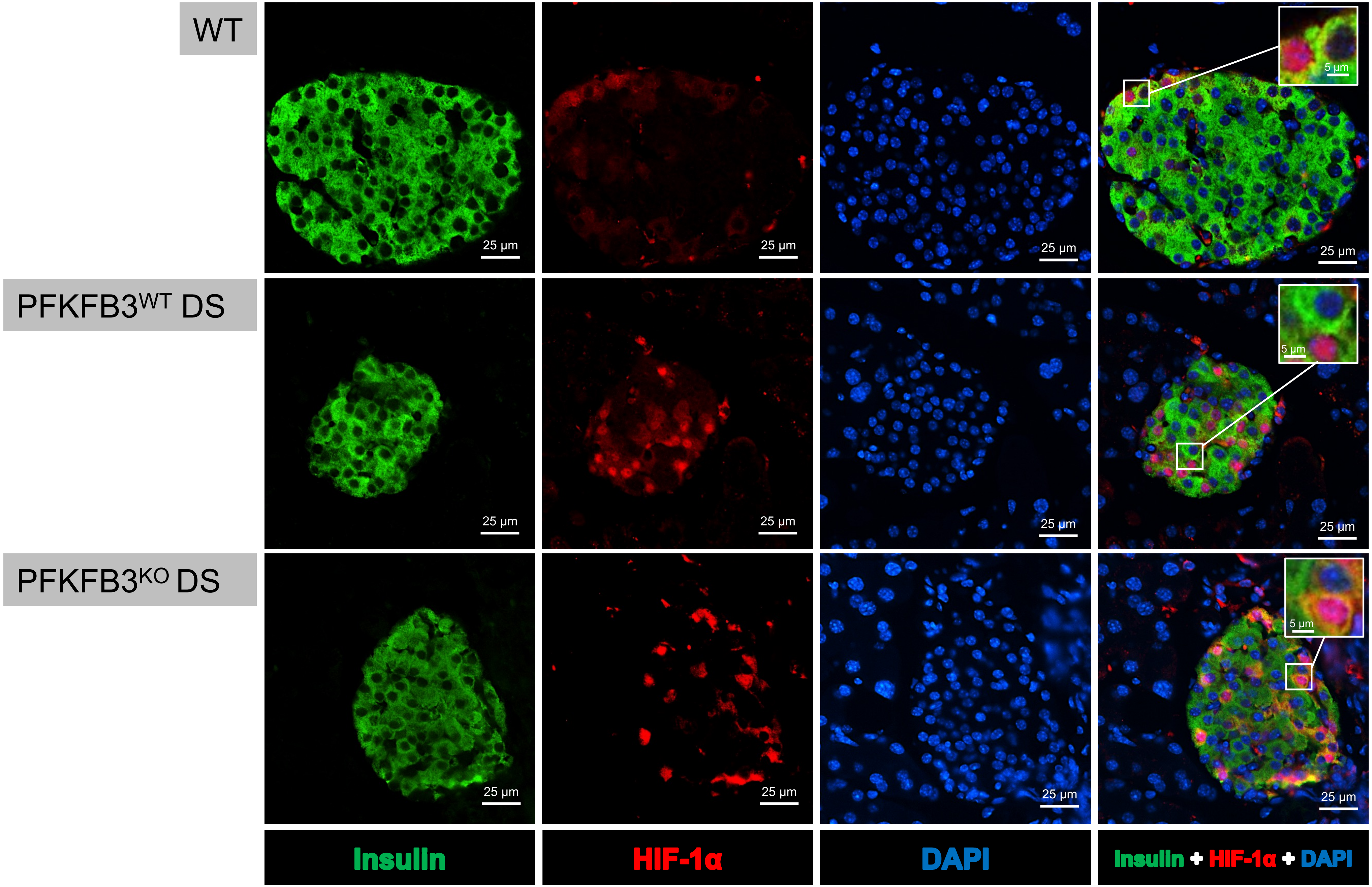

e. Supporting data for Figure 7a

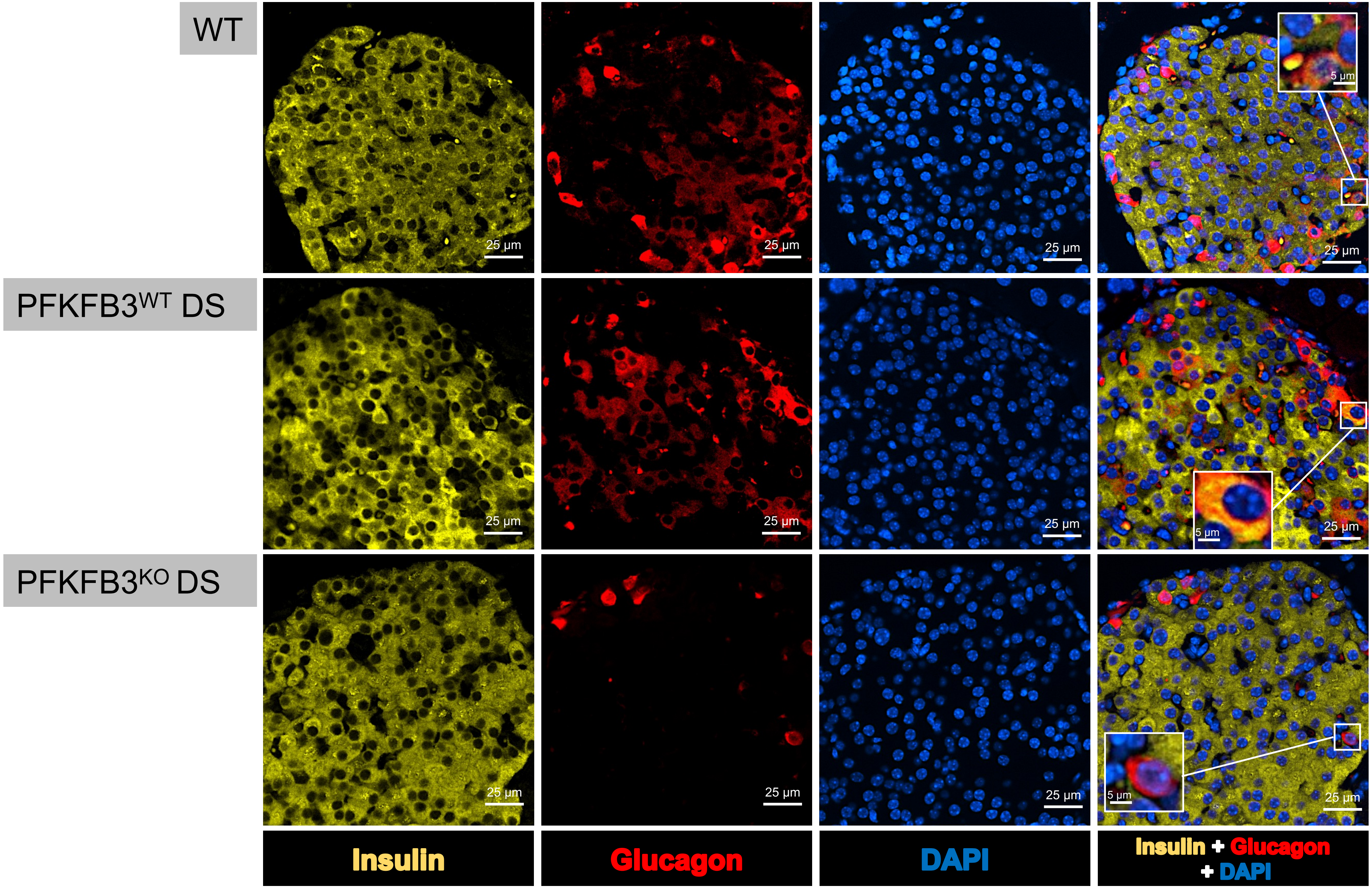

f. Supporting data for Figure 7a

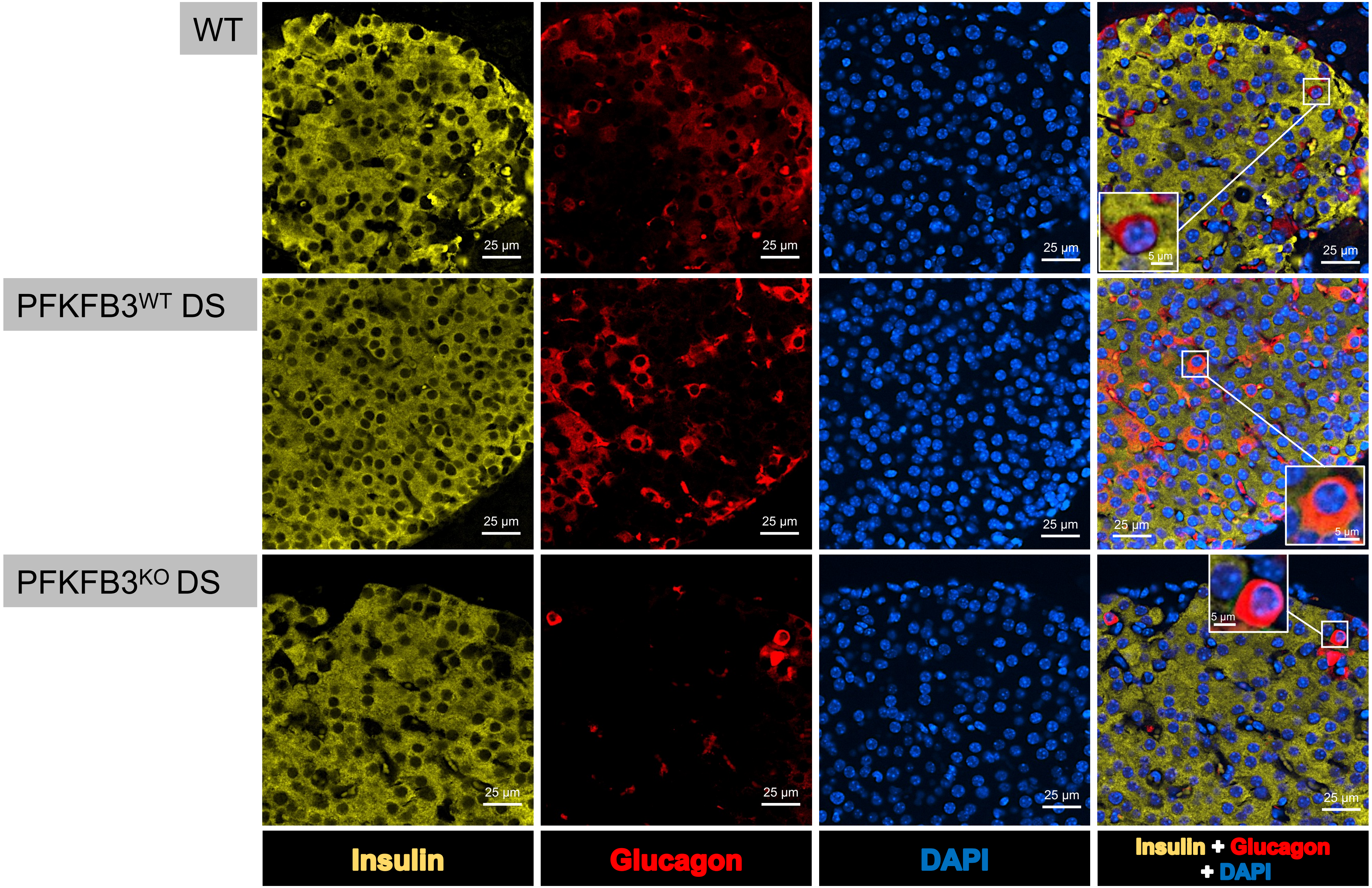

g. Supporting data for Figure 8c

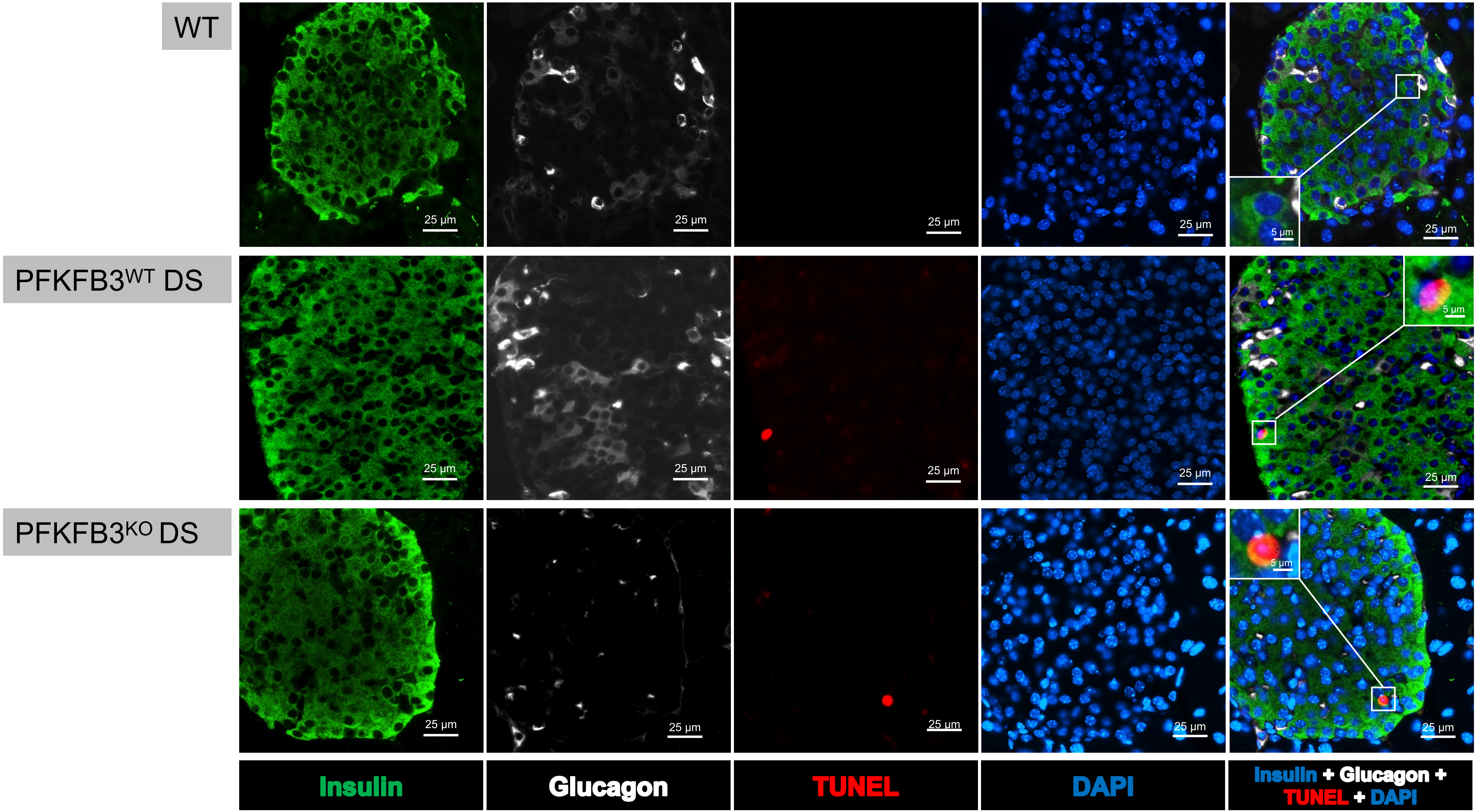

h. Supporting data for Figure 8e

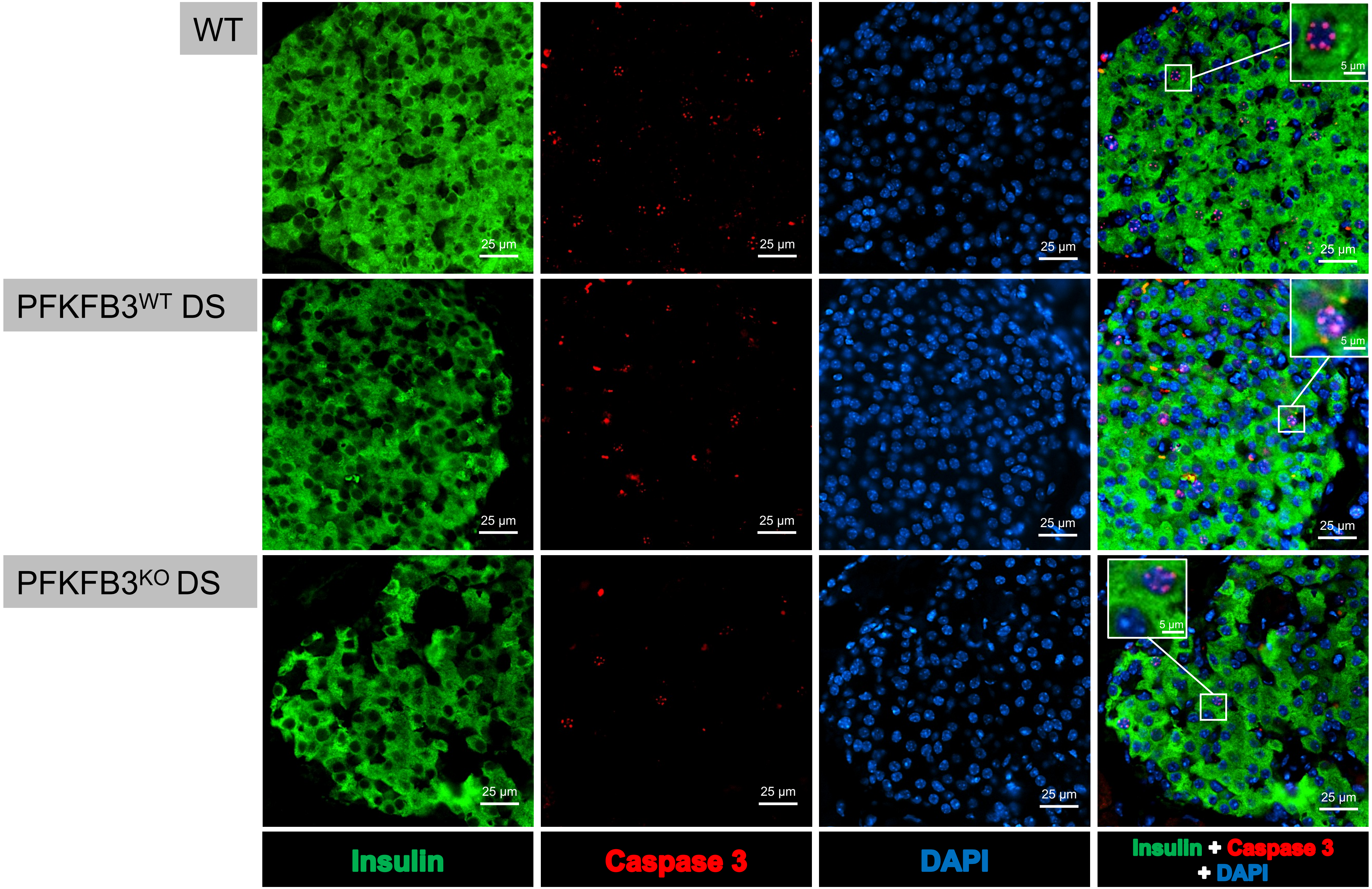

i. Supporting data for Figure 8e

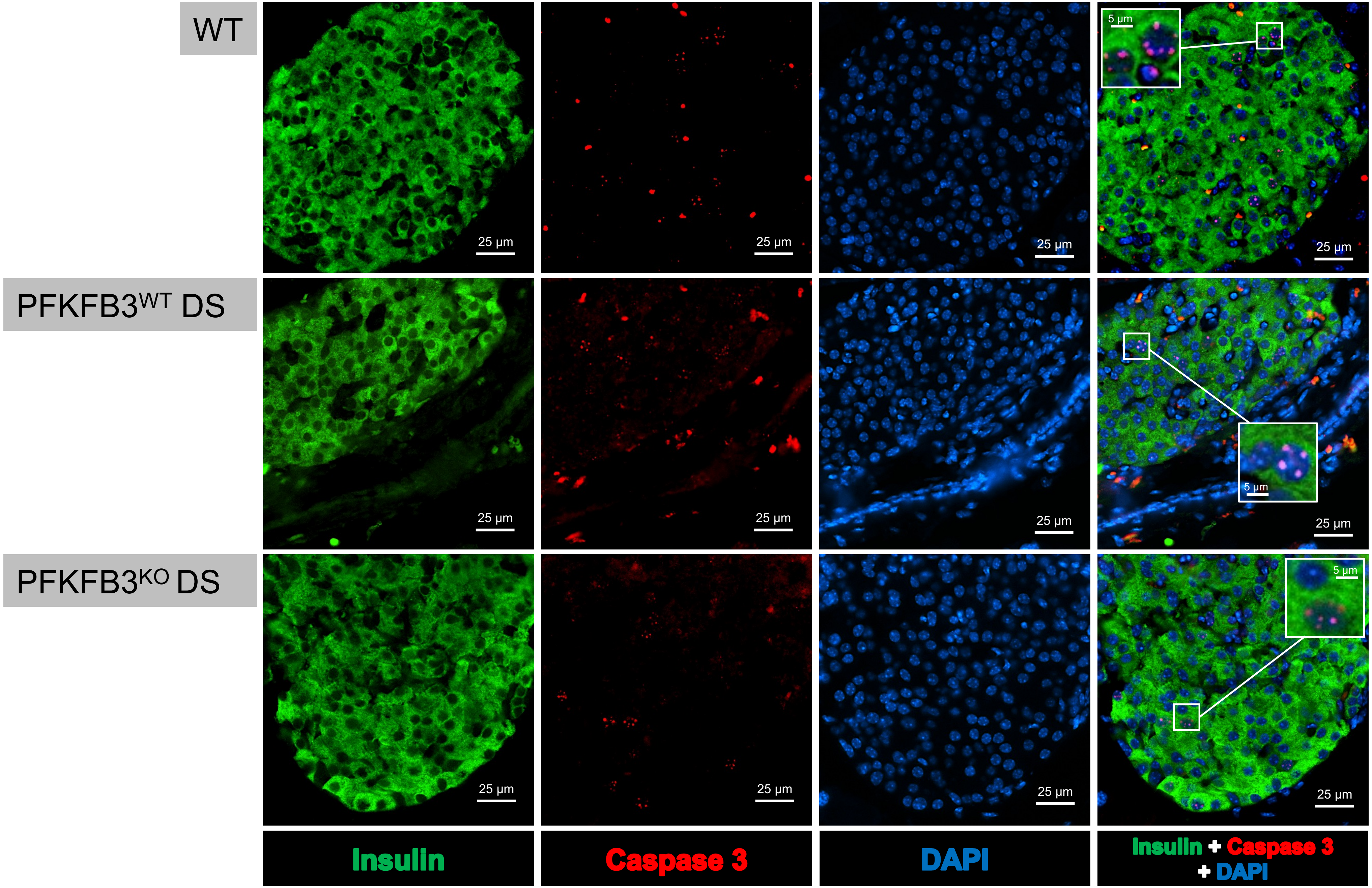

j. Supporting data for Figure 9a

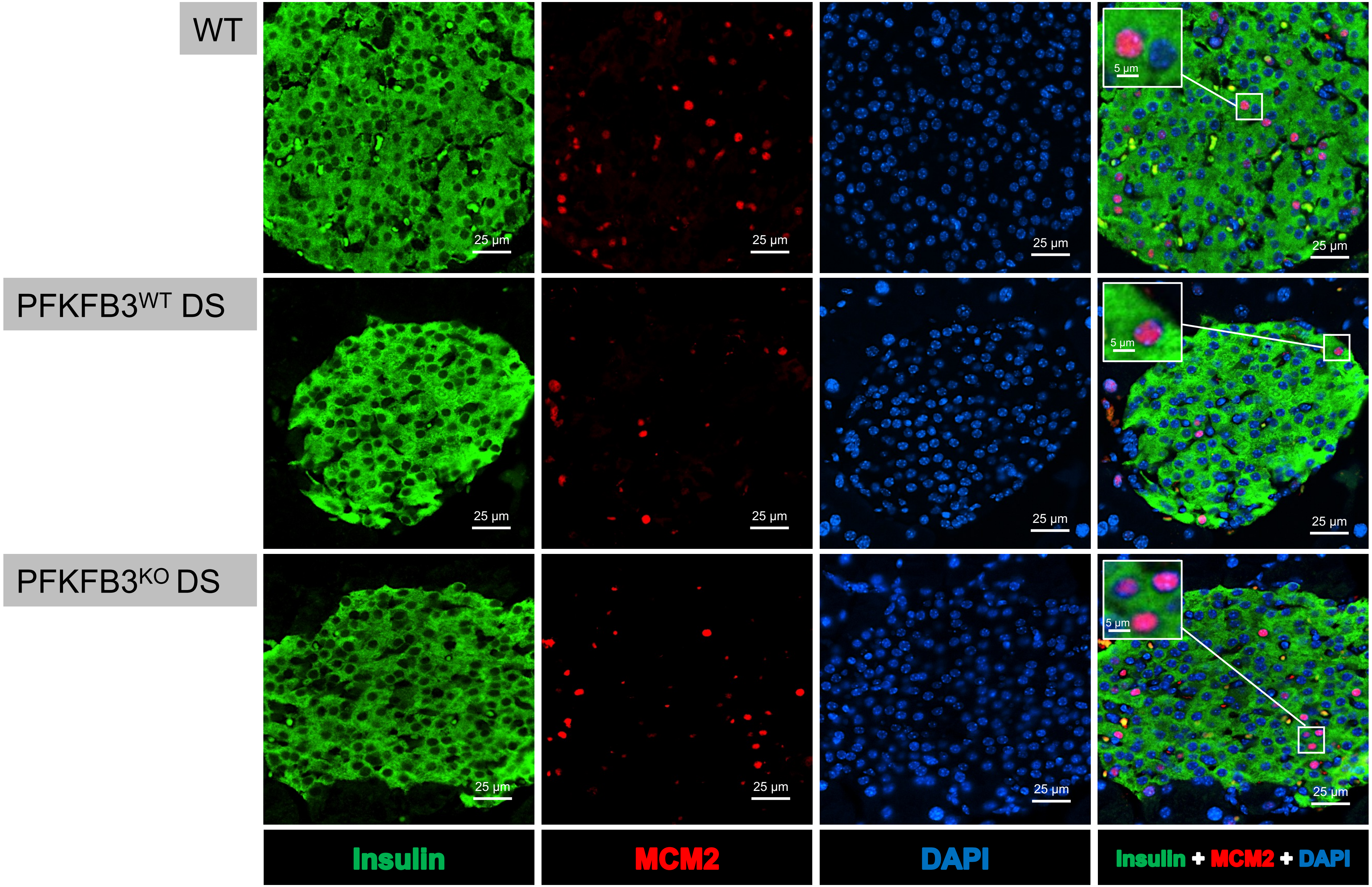

k. Supporting data for Figure 9a

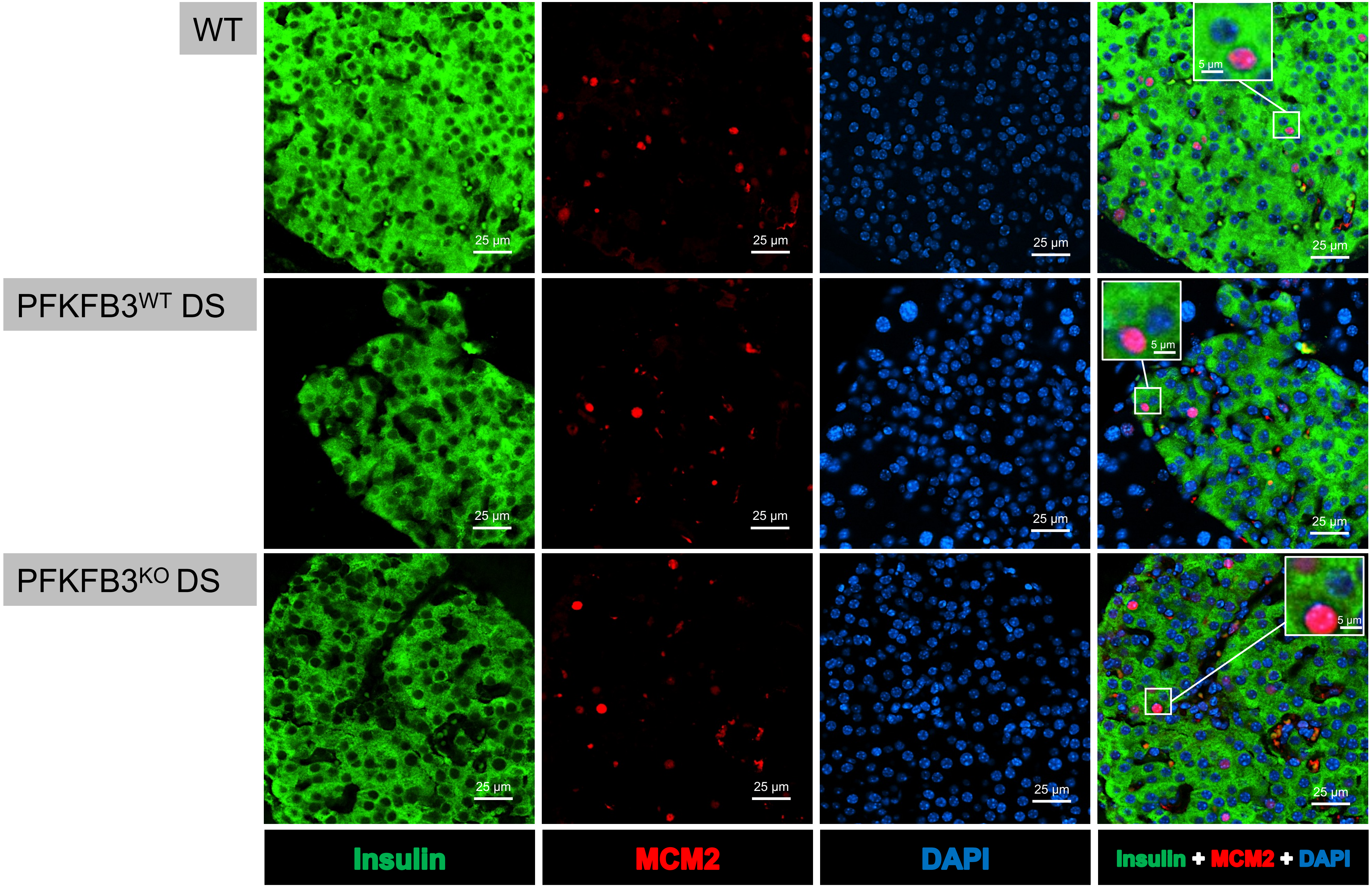

I. Supporting data for Figure 9c

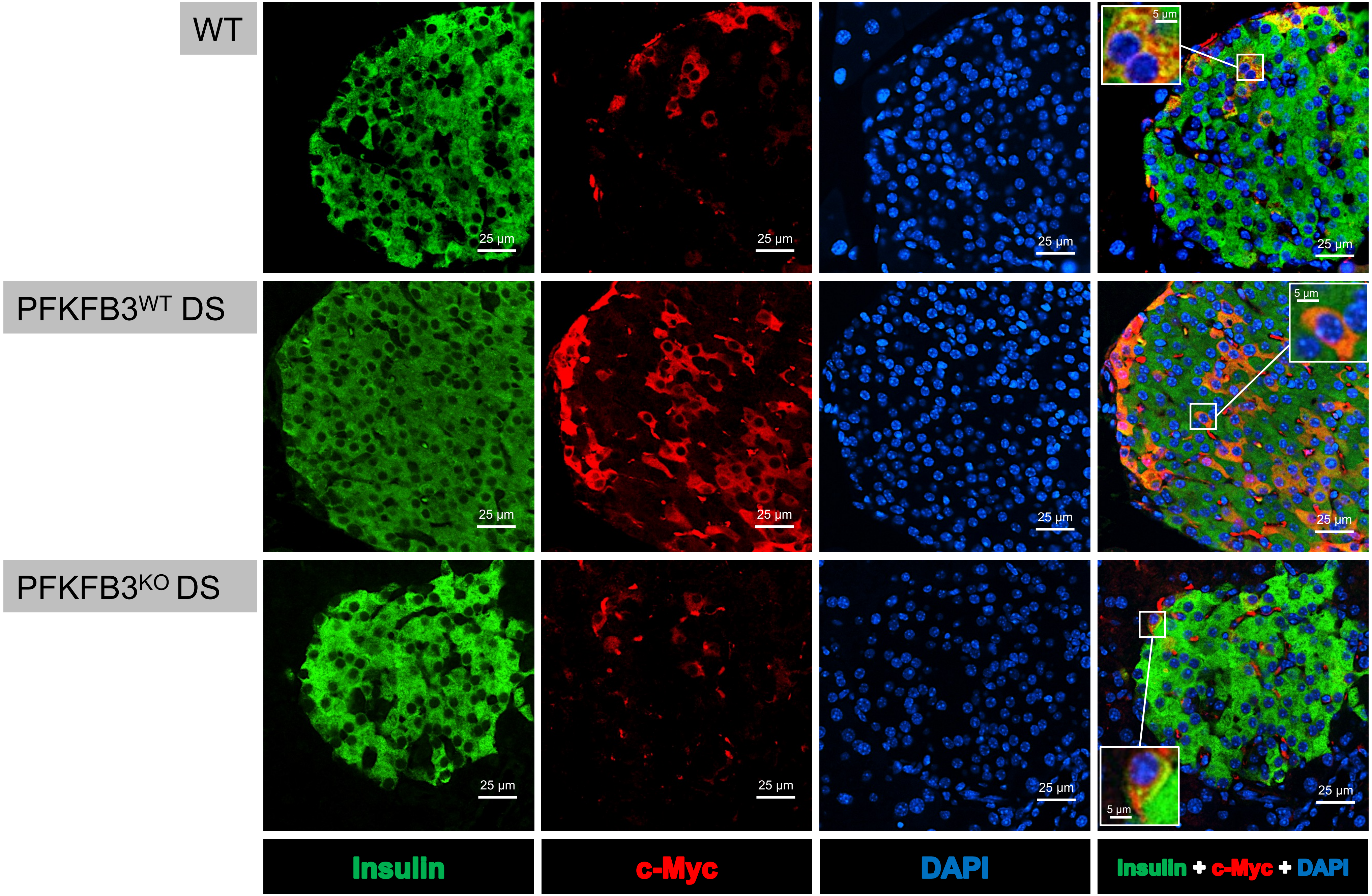

m. Supporting data for Figure 9c

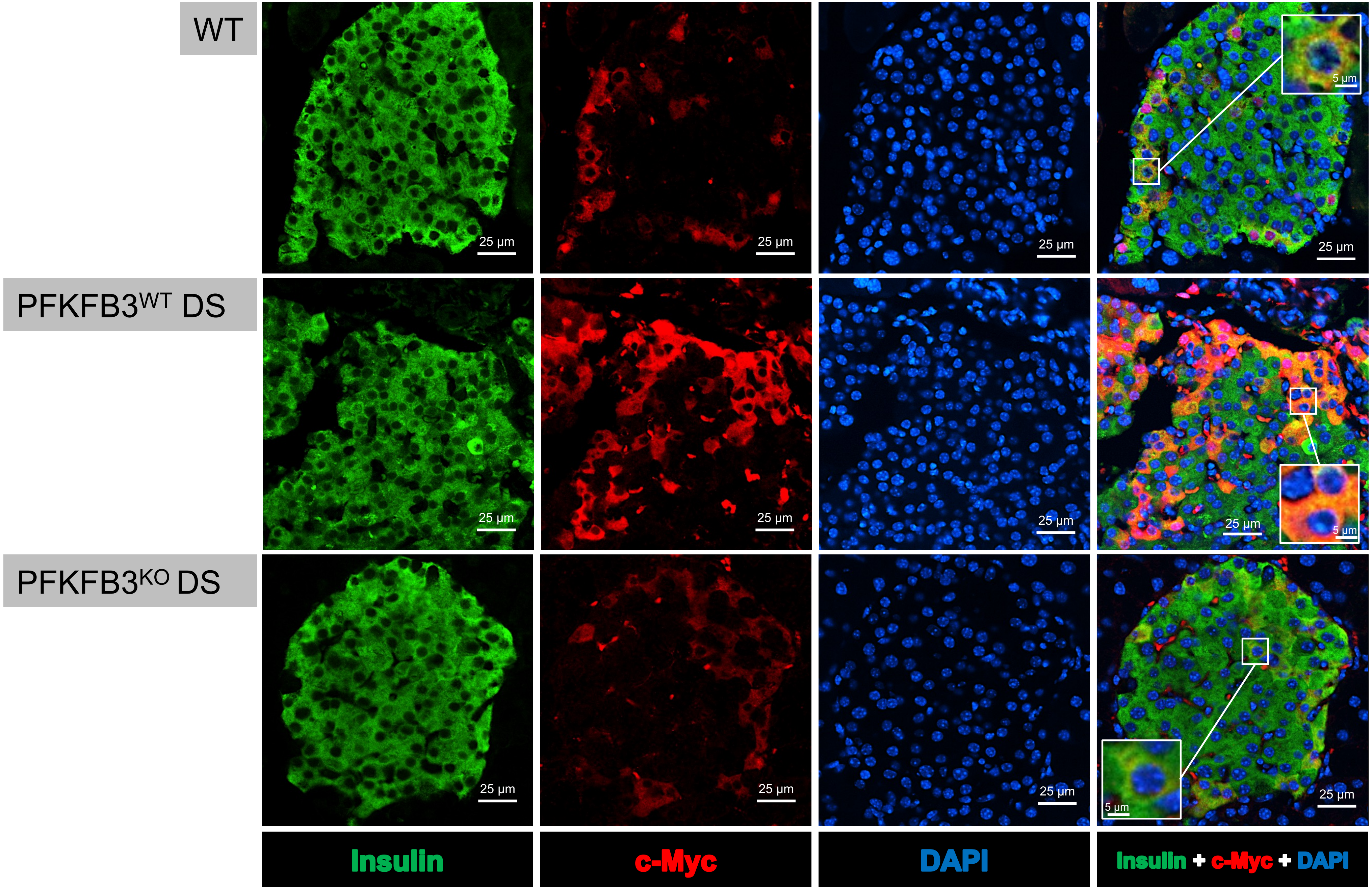

n. Supporting data for Supplementary Figure 13

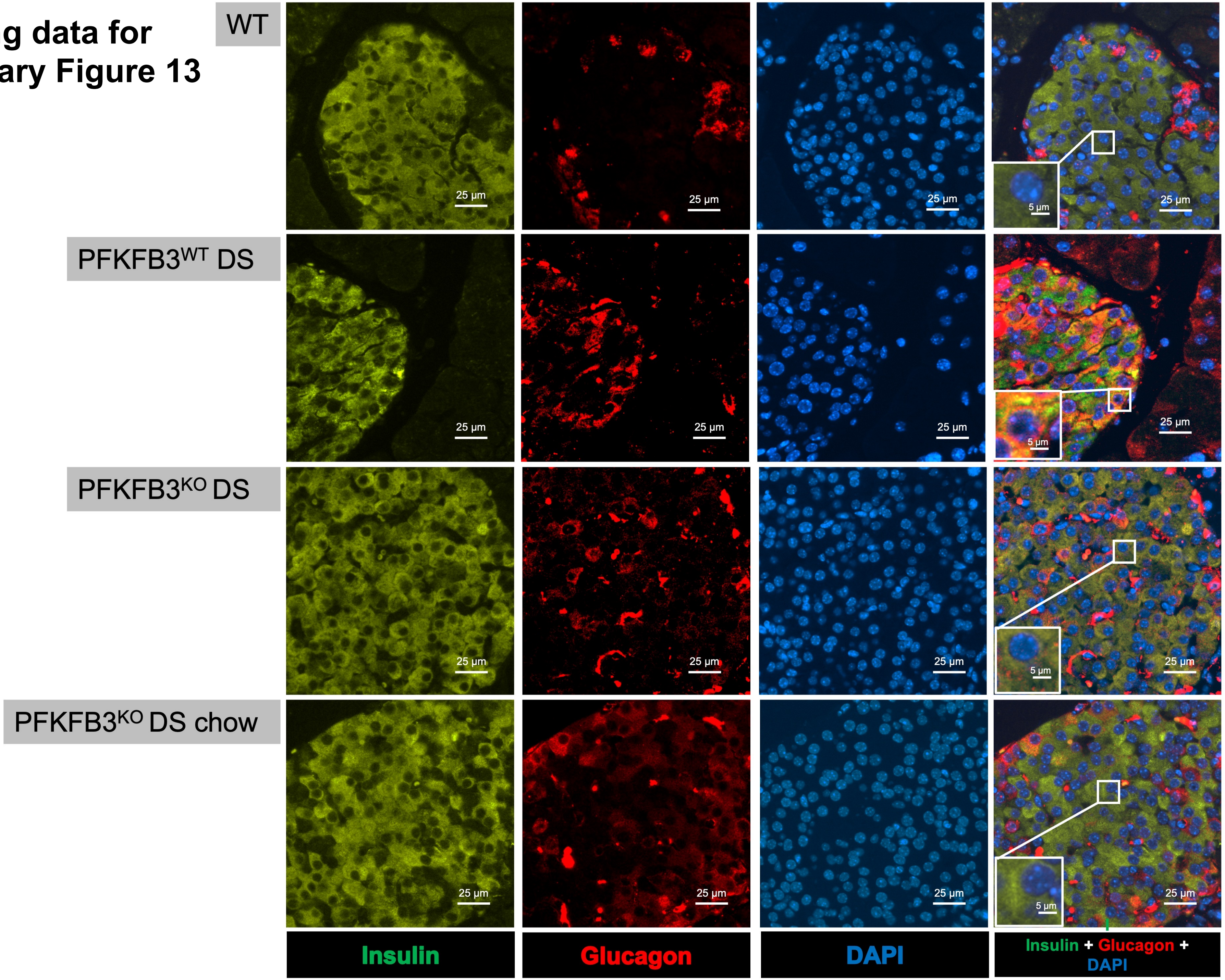

o. Supporting data for Supplementary Figure 15b

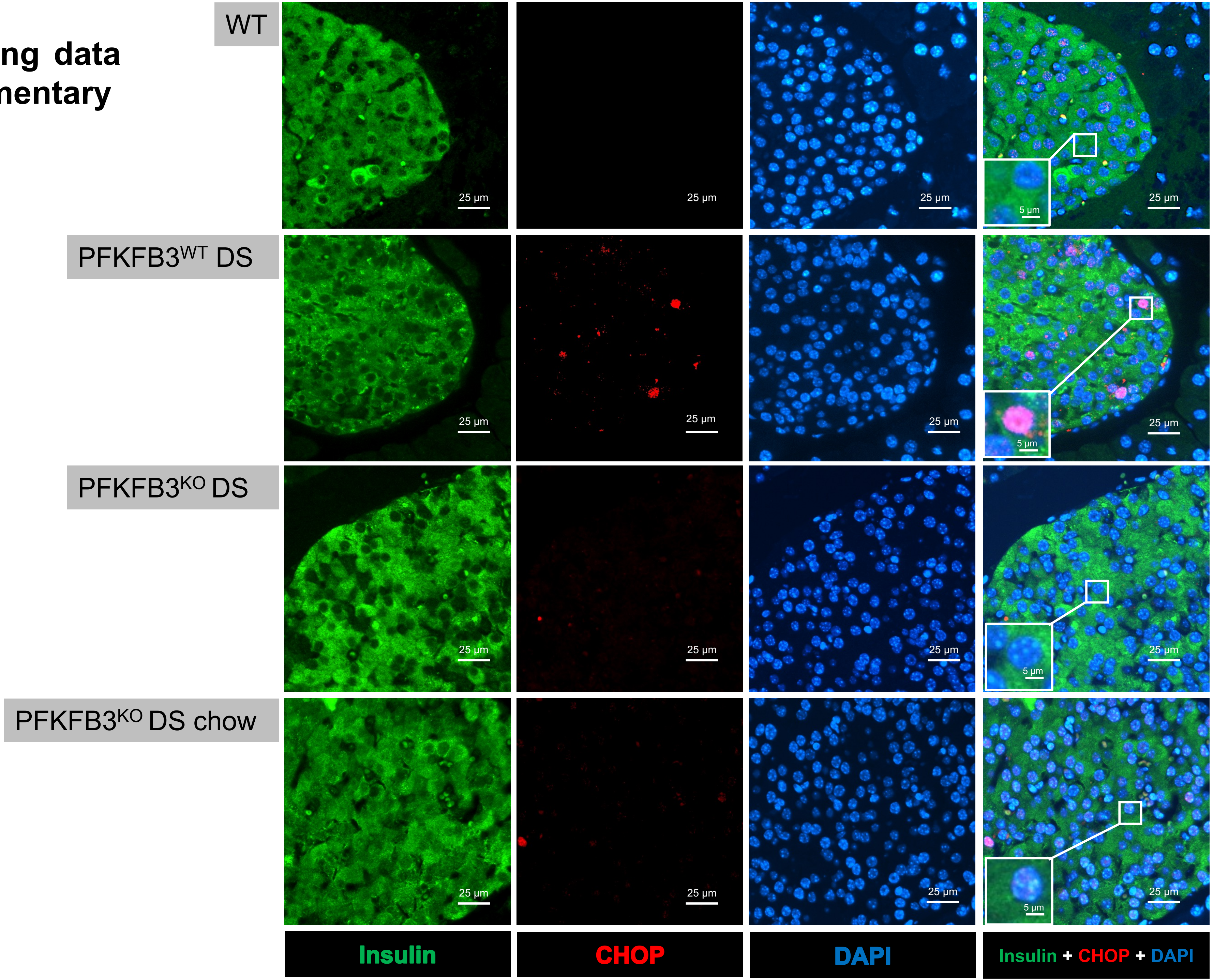

p. Supporting data for Supplementary Figure 16a

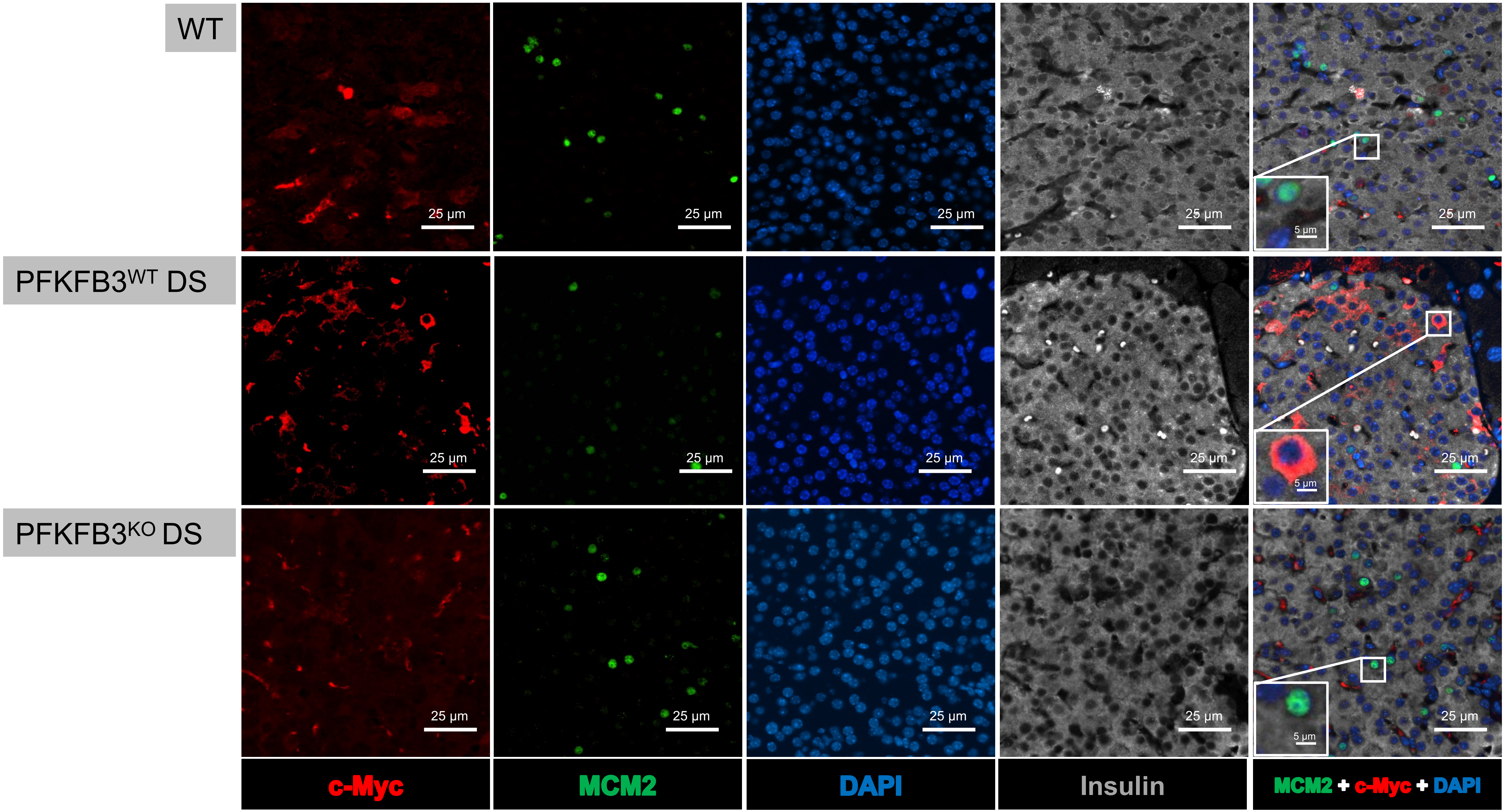

Supplementary Table 1 - LDHA-positive vs LDHA-negative β-cell comparison in T2D

	p_val	avg_logFC	pct.1	pct.2	p_val_adj
**LDHA**	1.06337848359989E-20	0.736410359051582	1	0	4.96523315347295E-16
**S100A10**	2.10940452800272E-09	0.530047408028363	0.679	0.078	9.8494425626031E-05
**SMIM24**	3.02392597221245E-08	0.595945390537383	0.679	0.094	0.00141196175420516
**TM4SF4**	3.32354002996006E-08	1.41226615308684	0.679	0.109	0.00155186054618925
**F10**	1.94744497323796E-07	0.395163750085695	0.643	0.109	0.00909320481354001
**GC**	1.99127816305625E-07	1.10304171584566	0.571	0.078	0.00929787512675855
**MYO10**	2.26920678648673E-07	0.39430141233161	0.536	0.062	0.0105956072481425
**USH1C**	5.17680944886665E-07	0.324829649353735	0.571	0.094	0.0241720763595931
**FXYD5**	6.94404590821441E-07	0.367562539993246	0.571	0.094	0.0324238335592255
**RGS4**	7.06217213950261E-07	1.29397621483838	0.679	0.234	0.0329754003709795
**ARX**	7.09227482273617E-07	0.207954231531124	0.464	0.047	0.033115958829802
**SPOCK3**	7.09558822131753E-07	0.346446498541144	0.393	0.016	0.033131430081798
**INS**	9.1592229249322E-07	-0.518250224744619	1	1	0.0427671596033859

Supplementary Table 2 - Cluster 1 comparison in non-diabetics vs T2D

	p_val	avg_logFC	pct.1	pct.2	p_val_adj
**SP100**	1.83529982769889E-15	0.378505445302907	1	1	8.56956548547445E-11
**PSPHP1**	2.20744542905235E-13	0.192776778346709	0.494	0.013	1.03072249418741E-08
**PRSS2**	4.30831031436233E-12	-0.162827815744324	0.053	0.347	2.0116793350852E-07
**ANXA2**	1.17918322353811E-11	-0.563211460832181	0.707	0.96	5.5059602256665E-07
**HLA-DQB1**	4.22582007470112E-09	-0.118541300184139	0.015	0.187	0.000197316216748019
**SELENOM**	4.57806548755217E-09	-0.334792388055403	0.57	0.867	0.000213763611810274
**RBP4**	1.41672401004439E-08	0.852515791041687	0.654	0.28	0.000661510942010028
**BSG**	1.47361893765802E-08	-0.385386528856361	0.981	0.987	0.00068807689056066
**SPP1**	3.93465927290205E-08	-0.925065270629486	0.209	0.52	0.00183721045429615
**SGIP1**	7.31732691792908E-08	-0.164998969371357	0.202	0.52	0.00341667945778862
**CBX6**	1.8497953593377E-07	0.309721421676018	0.973	1	0.00863724947135553
**RNF152**	2.52502071816576E-07	-0.161457893110474	0.163	0.427	0.0117900792393314
**DLK1**	2.6498653797298E-07	-0.850082485081069	0.635	0.827	0.0123730164175724
**SULF1**	4.26351994219233E-07	-0.144019985225868	0.152	0.413	0.0199076536660786
**CAPS2**	4.40191944204415E-07	-0.197951798845561	0.125	0.373	0.0205538824507367
**NEFL**	8.47960453055088E-07	-0.204532655298121	0.042	0.213	0.0395938174345012

Supplementary Table 3 - LDHA-positive β-cell comparison in non-diabetics vs T2D

	p_val	avg_logFC	pct.1	pct.2	p_val_adj
**RPS2P46**	5.85730011951328E-09	-0.1118549378086	0.925	0.964	0.000273494914480434
**PSPHP1**	2.70419201167337E-08	0.24418410322901	0.672	0	0.00126266837601065
**PRSS2**	1.90296200240872E-07	-0.322348427835777	0.03	0.464	0.00888550047784702
**CBX6**	9.49842470252106E-07	0.421997298176448	1	1	0.0443509944634816

Supplementary Table 4 – Enrich*r* pathway analysis of Cluster 7 vs Cluster 1 β- cells

a) **Non-diabetics**

**BioPlanet 2019**	**Wiki Pathways 2019**	**KEGG 2019 human**	**Elsevier Pathway Collection**	**Reactome 2016**	**TRRUST**
Metabolism	Cori Cycle	Circadian entrainment	L-cell: GCG, PYY, 5-HT release	Peptide hormone metabolism	HIF1α human
	Amino acid metabolism			Metabolism	

b) **T2D**

**BioPlanet 2019**	**Wiki Pathways 2019**	**KEGG 2019 human**	**Elsevier Pathway Collection**	**Reactome 2016**	**TRRUST**
Ghrelin synthesis and secretion	Coagulation	Coagulation	Ghrelin effect on insulin secretion	Synthesis, secretion and de-acetylation of ghrelin	JunB
Diabetes pathways	Cori Cycle	Insulin secretion		Peptide hormone metabolism	SP1

Supplementary Table 5 – Enrich*r* pathway analysis of LDHA-positive vs LDHA-negative β-cells

a) Non-diabetics

**BioPlanet 2019**	**Wiki Pathways 2019**	**KEGG 2019 human**	**Elsevier Pathway Collection**	**Reactome 2016**	**TRRUST**
TGFβ	Cori Cycle	Amino acid metabolism	Ghrelin effect on insulin secretion	Peptide hormone metabolism	HIF1α human
Metabolism	Amino acid metabolism	Insulin secretion	GCG and PPY regulation		SP4 human

b) T2D

**BioPlanet 2019**	**Wiki Pathways 2019**	**KEGG 2019 human**	**Elsevier Pathway Collection**	**Reactome 2016**	**TRRUST**
Insulin receptor substrate	Cori Cycle	HIF1α signaling	β - to α-cell conversion	Coagulation	DLX2
		Insulin secretion	Hypergly-cemia and hyperlipide-mia	IRS activation	ISL1

Supplementary Table 6 – Enrich*r* pathway analysis of differentially expressed genes between LDHA-positive vs LDHA-negative α-cells in health

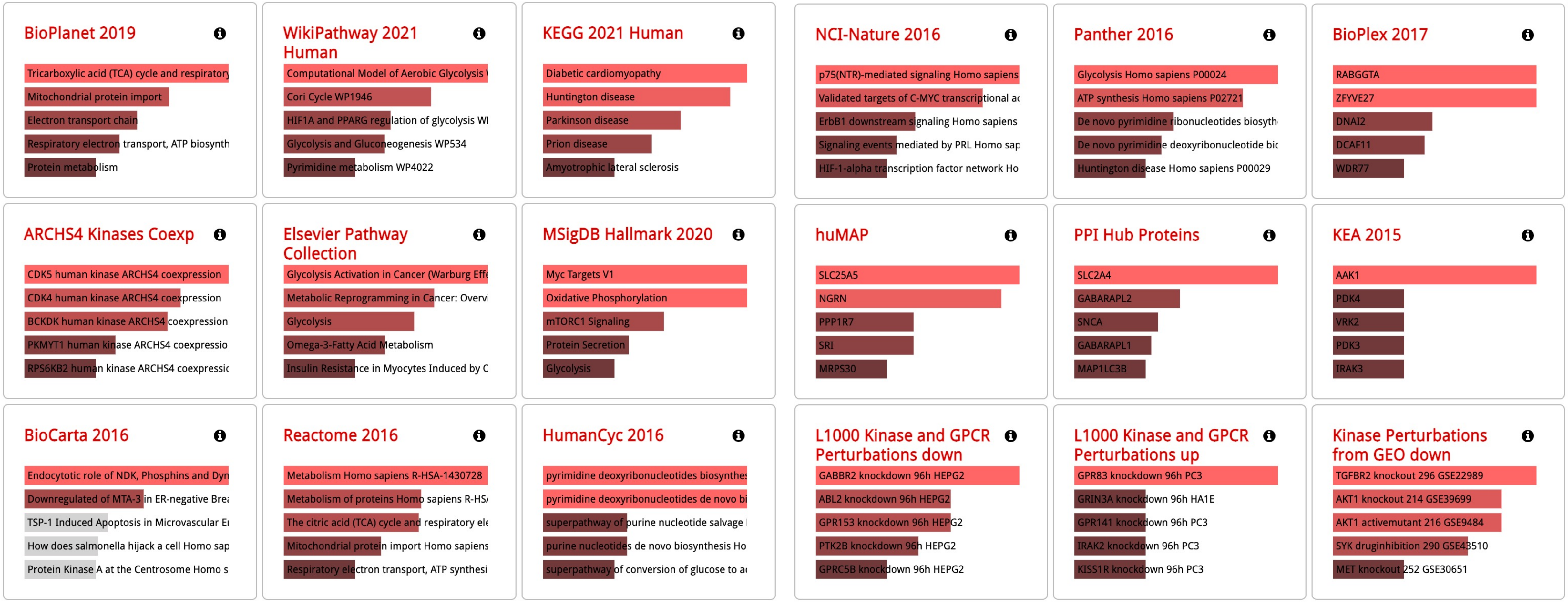

Supplementary Table 7 – Ingenuity pathway analysis

**Cluster 1 vs 7 in health**	**Cluster 1 vs 7 in T2D**	**LDHA pos vs neg in health**	**LDHA pos vs neg in T2D**
LXR/RXR activation	LXR/RXR activation	LXR/RXR activation	FXR/RXR activation
ILK signaling	FXR/RXR activation	FXR/RXR activation	Pyruvate to lactate
Cytotoxic T cell mediated apoptosis	Macro-pinocytosis	GPCR-mediated nutrient sensing	Extrinsic pro-thrombin activation
Opioid signaling	Insulin secretion	Neuropathic pain signaling	G protein signaling
Dopamine receptor signaling	Production of NOS and ROS in macrophages	Super-pathway of citruline metabolism	Coagulation

Supplementary Table 8 – plasma-to-whole blood glucose conversion coefficient derivation

Mouse ID	Body weight (g)	Whole blood glucose (mg/dl)	Plasma glucose (mg/dl)	Plasma/Whole blood glucose ratio
1618	23.9	162	333	2.055555556
1617	25.2	134	207	1.544776119
1616	25	95	212	2.231578947
1615	23.9	142	171	1.204225352
1608	29.4	115	199	1.730434783
1609	29.3	140	331	2.364285714
1610	27.5	109	227	2.082568807
1612	28.5	128	292	2.28125
1642	26.3	153	200	1.307189542
1643	28.8	102	205	2.009803922
1644	24.5	153	166	1.08496732
1657	22.6	156	261	1.673076923
1658	24.8	112	178	1.589285714
			Difference	1.781461438

Supplementary Figure Legends

Supplementary Fig. 1. ***Body weights of experimental groups.*** *Body weights in indicated experimental groups (**a**) at the baseline (t=0) (**b**) one week before start of HFD (**c**) after four weeks of HFD, and (**d**) after 13 weeks of HFD.*

Supplementary Fig. 2. ***Organ weights of experimental groups.*** *Weight (g) in indicated experimental groups (**a**) of pancreas (**b**) of liver and (**c**) of spleen.*

Supplementary Fig. 3. ***PFKFB3$^{\beta KO}$ DS mice demonstrate increased impairment of glucose tolerance at 13 weeks.***

*(**a**) Intraperitoneal glucose tolerance test (IP-GTT) at thirteen weeks post-onset of high-fat diet (HFD). (**b**) Quantification of the area under the curve (AUC) as mg/dl x min in the experimental groups shown in (a).*

Supplementary Fig. 4. **Quality of the cells from each donor.** *Violin plots showing the distribution of number of genes (**a**), number of transcripts (**b**) and percentage of mitochondrial expression (**c**) in the cells from each donor.*

Supplementary Fig. 5. **Quality of the cells from each cluster**. *Violin plots showing the distribution of number of genes (**a**) number of transcripts (**b**) and percentage of mitochondrial expression (**c**) in the cells from each cluster.*

Supplementary Fig. 6. ***Distribution of annotated pancreatic cell types in health versus T2D.*** *Relative contribution of nine annotated pancreatic cell types in health and T2D is presented as a percentage (%).*

Supplementary Fig. 7. ***Heat-map showing the top marker genes for each cluster.*** *The marker genes were ranked by expression fold changes to compare the indicated cluster with all the other clusters. The colour scale represents the scaled expression of the gene.*

Supplementary Fig. 8. ***Dot-plot showing the top marker genes for each cluster.*** *The marker genes were ranked by expression fold changes to compare the indicated cluster with all the other clusters. The size of the dots represents the percentage of cells in which the gene was detected. The colour scale represents the scaled expression of the gene.*

Supplementary Fig. 9. ***PFKFB3 expression in β-cells from sc RNA-Seq analysis.*** *(**a**) Expression levels of PFKFB3 in LDHA positive- (LDHA+) versus LDHA negative (LDHA-) β-cells in non-diabetics. (**b**) Expression levels of PFKFB3 in LDHA positive- (LDHA+) versus LDHA negative (LDHA-) β-cells in type-2 diabetics (T2D)*

Supplementary Fig. 10. β-cell cluster differences in ND and T2D from [27].

*STRING analysis was performed to present the relationship between the differentially expressed genes in cluster 7 versus cluster 1 (**a**) in health (ND) and (**b**) in type 2 diabetes (T2D).*

Supplementary Fig. 11. Differences in LDHA-positive versus LDHA-negative β-cells in ND and T2D. *We used STRING analysis to present the relationship between the differentially expressed genes [27] in LDHA-positive (cluster 7) and LDHA-negative (cluster 1) β-cells (**a**) in health (ND) and (**b**) in type 2 diabetes (T2D).*

Supplementary Fig. 12. β-cell differences in cluster 1 or LDHA-negative β-cells in healthy ND. *STRING analysis was performed to present the relationship between the differentially expressed genes [27] in health (ND) in either (**a**) cluster 1 or (**b**) LDHA-negative β-cells. No differences were observed relative to cluster 7 or to LDHA-positive β-cells.*

Supplementary Fig. 13. Double insulin- and glucagon positive bihormonal cells are reduced in PFKFB3^{βKO} DS mice at 8 weeks HFD *(**a**) Representative immunofluorescence images of islets from WT, PFKFB3^{WT} DS and PFKFB3^{βKO} DS mice immunostained for gucagon (red), insulin (green) and nuclei (blue).*

Supplementary Fig. 14. ***Bihormonal cells are associated with exposure to high fat diet*** *(**a**) Quantification of the ratio between bihormonal cells relative to all insulin positive cells (β- and bihormonal cells) (%) in indicated experimental groups. WT and homozygous hIAPP$^{+/+}$mice on chow diet with prediabetes (pre-DM) and diabetes (DM) (WT^{chow}, hTG-preDM and hTG-DM) were used for comparison to the study experimental groups. (**b**) Cell composition of β-, α-cells and bihormonal cells in indicated experimental groups (n=3, n=4 independent animals for PFKFB3$^{\beta KO}$ DS, SEM).*

Supplementary Fig. 15. ***At 8 weeks HFD, ER- but not IAPP-incurred stress is increased in PFKFB3^{WT} DS mice while PFKFB3$^{\beta KO}$ DS mice are ER-stress free*** *(**a**) Representative images from indicated experimental mouse groups at 8 weeks after HFD immunostained for cytoplasmic c-myc (red) and insulin (green). Last image represents PFKFB3^{WT} DS at 13 weeks used as internal positive control (**b**) Representative images from indicated experimental mouse groups at 8 weeks after HFD immunostained for CHOP (red) and insulin (green). (**c**) Quantification of CHOP positive β-cells in experimental groups as indicated in (b). (n=4 independent animals, SEM)*

Supplementary Fig. 16. ***Co-labelled MCM2- and cytoplasmic c-Myc positive cells rarely occur in any experimental group*** *(**a**) Representative images from indicated experimental mouse groups at 13 weeks after HFD co-labelled for MCM2 (green), cytoplasmic c-Myc (red) and insulin (grey). (**b**) Magnified sections from immunofluorescence images from a, co-labelled for MCM2 (green), cytoplasmic c-myc*

*(red) and insulin (grey) to demonstrate rarely observed overlap between the two markers. (**c**) Quantification of MCM2 positive β-cells in experimental groups as indicated in (a). (**d**) Quantification of cytoplasmic c-myc positive β-cells in experimental groups as indicated in (**a**). (n=3, n=4 independent animals for PFKFB3^{βKO} DS, SEM).*

<u>Supplementary Fig. 17.</u> Supporting image data for Figures and Supplementary Figures

*(**a-p**) Single channel and merged images used for the indicated Figures and Supplementary Figures and to demonstrate at least two most representative marker expression levels in the islets..*

Supplementary Tables

***Supplementary Table 1.** Differentially expressed genes in LDHA-positive versus LDHA-negative β-cells in T2D donors*

***Supplementary Table 2.** Differentially expressed genes in Cluster 1 β-cells in non-diabetic versus T2D donors*

***Supplementary Table 3.** Differentially expressed genes in LDHA-positive β-cells in non-diabetic versus T2D donors*

Supplementary Table 4. *Summary of Enrichr analysis of differentially expressed genes in Cluster 7 versus Cluster 1 β-cells in health and T2D*

Supplementary Table 5. *Summary of Enrichr analysis of differentially expressed genes in LDHA-positive versus LDHA-negative β-cells in health and T2D*

Supplementary Table 6. *Summary of Enrichr enrichment analysis across the curated libraries*

Supplementary Table 7. *Summary of of Ingenuity Pathway analysis of differentially expressed genes in Cluster 7 versus Cluster 1- and LDHA-positive- versus LDHA-negative β-cells in health and T2D, respectively*

Supplementary Table 8. *Plasma- and whole blood glucose measurements used to derive a conversion coefficient*
