# Supplementary material for: β-cell-specific deletion of PFKFB3 restores cell fitness competition and physiological replication under diabetogenic stress
Source: Commun Biol. 2022 Mar 22;5:248. doi: 10.1038/s42003-022-03209-y (PMC8941137; doi:10.1038/s42003-022-03209-y)
Supplement: Supplementary file 3 — Description of Additional Supplementary Files [file 42003_2022_3209_MOESM3_ESM.pdf]

Supplementary Data Files

File name: Supplementary Data File 1.

Description: *Differentially expressed genes in Cluster 1 versus Cluster 7 β-cells in both health and T2D*

File name: Supplementary Data File 2.

Description: *Differentially expressed genes in Cluster 1 versus Cluster 7 β-cells in non-diabetic donors*

File name: Supplementary Data File 3.

Description: *Differentially expressed genes in LDHA-positive versus LDHA-negative β-cells in non-diabetic donors*

File name: Supplementary Data File 4.

Description: *Differentially expressed genes in Cluster 1 versus Cluster 7 β-cells in T2D donors*

File name: Supplementary Data File 5.

Description: *Differentially expressed genes in LDHA-positive versus LDHA-negative α-cells in (**a**) non-diabetic- and (**b**) T2D donors*

File name: Supplementary Data File 6.

Description: *Original single values from each measurement used for generation of the graphs in the manuscript*
